# Analysis of the Differences Resulting from the Determination of Langmuir Isotherm Coefficients from Linear and Non-Linear Forms—A Case Study

**DOI:** 10.3390/ma18153506

**Published:** 2025-07-26

**Authors:** Joanna Lach

**Affiliations:** Faculty of Infrastructure and Environment, Czestochowa University of Technology, Brzeźnicka 60a, 42-200 Częstochowa, Poland; joanna.lach@pcz.pl; Tel.: +48-34-32-50-917

**Keywords:** Langmuir isotherms, lineal and non-linear forms, statistical measures

## Abstract

The sorption process is most commonly described by Langmuir isotherms, which can be calculated from either a non-linear form or various linear forms. Despite the fact that the non-linear model is now preferred, articles using linear models continue to be submitted to journals. On the basis of 68 isotherms, it was found that the linear Hanes–Woolf model (the most commonly used) gives the most similar q_m_ and K_L_ values to the non-linear model. The largest differences were obtained by determining the isotherm from the non-linear and linear forms of the Lineweaver–Burk model (this is the model often used by researchers). The evaluation of isotherms should not be performed solely on the basis of the coefficient of determination R^2^, which was intended for linear equations. Statistical measures such as the mean relative error, sum of squares of errors, chi-square statistic, sum of absolute errors, hybrid fractional error function, mean squared error were analysed. On the basis of the coefficient of determination, the Hanes–Woolf linear model was found to best describe the actual results, and on the basis of the other statistical measures, the isotherm determined from the non-linear form was found to be the best fit for the study.

## 1. Introduction

Many different isotherm models describing the adsorption process can be found in the literature, based on the work of authors such as Langmuir, Freundlich, Sips, Toth, Jovanovic, Tempkin. Elovich, Dubinin–Radushkevich, and Redlich–Peterson [1,2,3,4]. Langmuir and Freundlich adsorption isotherms are most commonly used to describe adsorption from aqueous solutions. In many articles, the results are described only with these two models [5,6,7,8]. Even when several adsorption models are used in a paper, it is almost always the Langmuir and Freundlich models that are placed first in the description and tables as the primary ones. These isotherms are compared with each other mainly on the basis of the coefficient of determination R^2^ [9,10,11]. Less frequently, other types of errors have been used to assess these models [12,13]. However, it should be stressed that the coefficient of determination has its limitations and basing one’s conclusions solely on this coefficient is not always appropriate. Another problem encountered in the determination of isotherm coefficients is how to determine them. Nowadays, in the age of ubiquitous computerisation, it would seem that calculating these coefficients from non-linear forms should present no difficulties and should be the only valid way to determine these coefficients. Nevertheless, based on personal experience, I know that in many cases isotherm coefficients are still determined from their non-linear forms. Of the 14 articles I reviewed in 2024, in 9, isotherm coefficients were determined from their linear forms. These articles were submitted to journals with Impact Factor (IF) values ranging from 2.3 to 4.4. Of course, all these articles, in their final version, had these isotherms calculated from a non-linear version. However, it is now equally possible to find published articles that have these values calculated from the linear version [14,15,16,17,18,19,20].

The problem of using linear forms is more prevalent for the Langmuir isotherm and less so for the Freundlich isotherm. The linear form of the Freundlich isotherm was determined by a mathematical transformation of this isotherm, while there are several models of the linear form of the Langmuir isotherm. Thus, what we have here is a “superimposition” of the linear model on the Langmuir isotherm model. Also relevant to the results is which of these models will be used to calculate the Langmuir isotherm coefficients. Six linear forms of the Langmuir isotherm can be found in scientific articles [21,22], but four of them are mainly used in scientific articles [23,24]. Usually, however, authors describe their research using only one non-linear form, without justifying why this particular form was chosen to describe the research results [25,26,27,28].

The Langmuir isotherm is often used to compare the sorption properties of different adsorbents. Mainly, the factor q_m_, which determines the maximum capacity of a monolayer, is used for comparison. Many authors of research papers tabulate a comparison between the sorbents used in the study and those reported in the literature [29,30,31]. Usually, when comparing q_m_, authors do not pay attention to how other researchers have determined this parameter. Only in exceptional cases do the authors of such compilations specify whether the isotherm was determined from a linear or non-linear form, but even so, they usually do not specify which linear form was used for the calculations [12]. This raises the question of whether the determination of these values from the different linear and non-linear forms will greatly affect the q_m_ values obtained. In addition, many articles include a comparison of Freundlich and Langmuir isotherms based on R^2^ [32,33]. This raises a further question as to whether the comparison of these isotherms determined from the linear forms can affect the errors in the conclusions.

For many years now, authors have been signalling the problem associated with the inaccuracy of determining Langmuir isotherm coefficients from linear forms [34]. However, the authors of these articles often evaluate only one linear form and compare it with a non-linear form [35,36] or evaluate the fit of isotherm forms to the test results, for example, only on the basis of the coefficient of determination R^2^ [37,38]. What is lacking is a comprehensive evaluation of the various linear and non-linear forms for many sorbents and sorbates using various statistical measures. In addition, the size of the difference occurring between q_m_ and K_L_ values calculated from linear and non-linear Langmuir forms is an interesting problem.

The aim of this study is to compare Langmuir isotherm coefficients determined from the non-linear form and various linear forms. The comparison will concern both the values of the Langmuir isotherm constants q_m_ and K_L_, the magnitude of the determination coefficients R^2^ and other statistical measures: average relative error, sum of squares of errors, chi-squared statistics, the sum of the absolute errors, the hybrid fractional error function, and the root mean square error. In addition, the Freundlich isotherms determined from the non-linear form and the Langmuir isotherms determined from the non-linear form and four different linear forms will be compared with each other on the basis of the R^2^ coefficient. These comparisons will be made on the basis of the author’s research data on the adsorption of both organic and inorganic compounds on different activated carbons. The results presented in the paper concern a large number of isotherms representing the adsorption of various adsorbates (organic and inorganic) on several activated carbons. In addition, an evaluation of these isotherms was carried out using a large number of statistical measures. Such comprehensiveness of the approach to the subject under discussion distinguishes this article from many already published scientific articles and allows some generalisation of conclusions.

## 2. Research Methodology

### 2.1. Material Used in the Analysis

This research used the results of our own research, which was published in the last 5 years. In the published articles, the Langmuir and Freundlich isotherms were determined from the non-linear Equations (1) and (2) and were mostly based on the R^2^ coefficient only. These articles describe the sorption of organic and inorganic compounds.

(1) $$q=\frac{q_{m}\cdot K_{L}\cdot C_{e}}{1+K_{L}\cdot C_{e}}$$

(2) $$q=K_{F}\cdot C_{e}^{\frac{1}{n}}$$

Commercial and modified activated carbons (ACs) were analysed in the study. Adsorption measurements were carried out at different temperatures and solution pH values. The articles [39,40,41,42,43] present the sorption results and Table 1 presents the highlights of the studies presented in these articles.

In the articles presented above, there were 68 adsorption isotherms for which further calculations were performed.

For the initial data described in Section 2.1, isotherms were determined from four linear forms of the Langmuir model (3)–(6) [44,45,46]. All measurement points were used to determine them, without discarding any points due to their large impact on the analysed errors or the obtained q_m_ and K_L_ values.

Langmuir form I (Lineweaver–Burk)—the q_m_ and K_L_ constants were calculated from the relation 1/q of 1/C_e_

(3) $$\frac{1}{q}=\left(\frac{1}{K_{L}\cdot q_{m}}\right)\frac{1}{C_{e}}+\frac{1}{q_{m}}$$

Langmuir form II (Hanes–Woolf)—the q_m_ and K_L_ constants were calculated from the relation C_e_/q of C_e._

(4) $$\frac{C_{e}}{q}=\left(\frac{1}{q_{m}}\right)\cdot C_{e}+\frac{1}{K_{L}\cdot q_{m}}$$

Langmuir form III (Edie-Hofstee)—the q_m_ and K_L_ constants were calculated from the relation q of q/C_e_

(5) $$q=\left(\frac{-1}{K_{L}}\right)\frac{q}{C_{e}}+q_{m}$$

Langmuir form IV (Scatchard)—the qm and KL constants were calculated from the relation q/C_e_ of q.

(6) $$\frac{Q}{C_{e}}=\left(-K_{L}\right)q+K_{L}\cdot q_{m}$$

### 2.2. Statistical Analysis

The statistical errors shown in Table 2 were used to assess the fit of the isotherms to the test results obtained. These are the error measures most commonly used in the literature for evaluating adsorption isotherm models. The formulas in Table 2 were used to calculate errors from both linear and non-linear forms.

## 3. Results and Discussion

### 3.1. Analysis of q_m_ and K_L_ Values of the Langmuir Isotherm

Next to the Freundlich isotherm, the Langmuir isotherm is the most commonly used to describe the results of adsorption studies. One of the advantages of this isotherm is that it is possible to determine precisely the coefficient q_m_, which determines the capacity of the monolayer. This coefficient gives the maximum adsorption capacity of a given compound, assuming that a single adsorption layer is formed. The q_m_ value, given, e.g., in mg/g or mmol/g, is readily used to compare, e.g., different sorbents, to assess the effect of pH and temperature on adsorption effects or to evaluate the effects of sorbent modifications. The constant q_m_ can also be used, for example, to calculate the specific surface area of a sorbent. Many authors use the q_m_ value to compare the properties of sorbents with other researchers [51,52,53]. The question is whether these values can be compared if they have been calculated from the non-linear form (1) or from the different linear forms (3)–(7). The linear forms of the Langmuir isotherm are not a mathematical transformation of the isotherm equation, as is the case with the linear form of the Freundlich isotherm. It is, therefore, a certain superposition of various linear models on the Langmuir isotherm model. It should also be noted that different authors use different linear forms of the Langmuir isotherm to calculate constants. In an attempt to assess the differences resulting from the way in which the Langmuir isotherm constants are calculated, the coefficients from the non-linear form and the four linear forms were determined. The Langmuir isotherms calculated in this way are included in the supplement in Tables S1–S11. The 68 isotherm constants are included in the tables, with published values marked with “*”(constants calculated from non-linear forms).

Table S1–S11 summarises the values of the capacity quotient of the monolayer calculated from the quotient of the linear forms and the non-linear form (q_ml_/q_mn_). Whereby q_mn_ denotes the value calculated from the non-linear equations and q_mI_ denotes the value calculated from the linear forms. Table 3 already shows only the range of values obtained, and the mean, median, and number of quotients of q_ml_/q_mn_ ≥ or < from 1 (detailed data in Tables S1–S11). For each of the linear forms, the monolayer capacity results obtained are both smaller and larger than those obtained from the non-linear form. The smallest differences were observed when the constant q_mI_ was calculated from linear form II (Hanes–Woolf). This is the form most commonly used by researchers to determine constants from the linear form. The ratio q_mI_/q_mn_ ranged from 0.93 to 0.10, the arithmetic mean was 1, and the median was 0.99. The q_m_ values obtained from linear form II (4) differed little from those obtained from the non-linear form. Only slightly larger differences were obtained between the constants calculated from linear form III and from the non-linear form. The greatest differences in q_m_ values were observed when the constants were calculated from the I linear form of the Langmuir isotherm (3). For this form, the ratio q_mI_/q_mn_ ranged from 0.83 to 1.75. For the I form of the isotherm, the highest arithmetic mean of 1.09 and median of 1.08 were also obtained.

Considering the frequency of use of linear forms for the calculation of Langmuir isotherms, it should be noted that linear form II is used most frequently. Less frequently, but also frequently, linear form I is used, for which the differences obtained between the constants calculated from the non-linear form are the highest. Forms III and IV are used very infrequently, mainly when authors compare multiple linear forms with each other.

**Figure 1:**
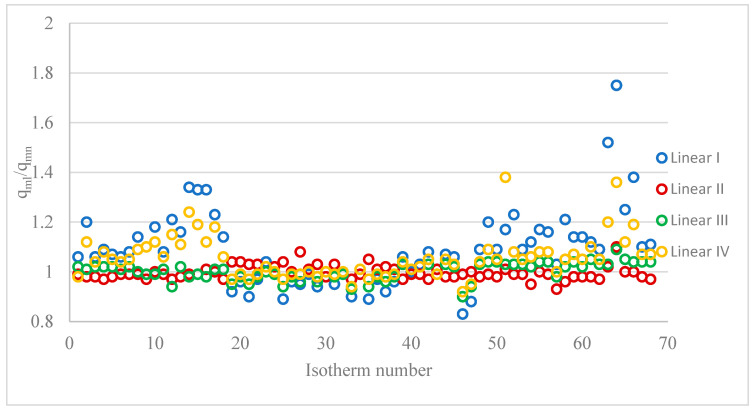
Values of q_ml_/q_mn_ for 68 isotherms and different linear forms.

Table 3 also shows the number of isotherms for which the obtained q_m_ values from the linear equations are less than, equal to, or greater than the constants determined from the non-linear forms (q_ml_/q_mn_ < 1; q_ml_/q_mn_ = 1; q_ml_/q_mn_ > 1). Figure 1 shows the q_ml_/q_mn_ values obtained (based on the data presented in Tables S1–S11). It was found that it was not possible to determine unambiguously that the obtained q_m_ values from the linear form would be greater or less than those obtained from the non-linear form. When q_m_ was calculated from the II linear form, in 40 cases (58.8%), q_m_ values higher than those calculated from the non-linear form were obtained, and in 19 cases (28.0%), the obtained q_m_ values were lower. When the other non-linear forms of the Langmuir isotherm were used, most of the q_m_ values were lower than the values obtained from the non-linear form.

**Figure 2:**
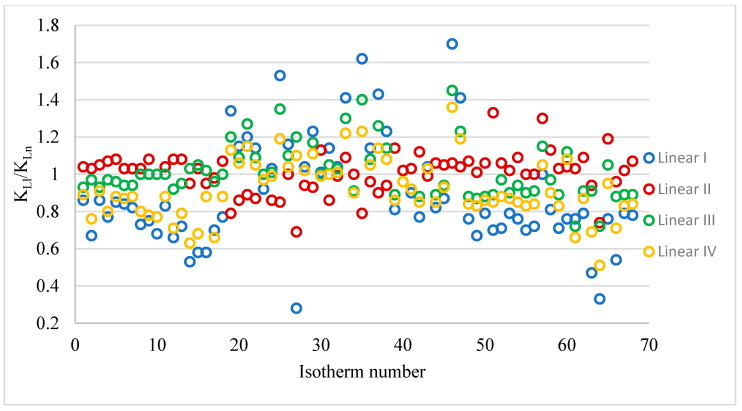
Values of K_Ll_/K_Ln_ for 68 isotherms and different linear forms.

All values of the K_L_ constant and the K_LI_/K_Ln_ quotients obtained from the linear and non-linear forms are provided in the supplement in Tables S1–S11. Table 4 summarises the K_LI_/K_Ln_ results giving the range of quotient values, arithmetic means, medians and the number(percentage) of K_LI_/K_Ln_ <, =, > values from 1. Figure 2 shows the K_LI_/K_Ln_ quotient values for the different linear forms. The obtained K_L_ values from the linear equations differ to a much greater extent from those values obtained from the non-linear equations than was observed for the constant q_m_. The smallest differences between the K_L_ values calculated from the linear and non-linear equations were obtained for the linear equation form II (range K_LI_/K_Ln_ 0.69–1.33). For this linear form, an arithmetic mean close to unity (1.01) and a median of 1.03 were obtained. The largest differences were obtained when the K_L_ constants were calculated from the I linear form of the Langmuir isotherm. In this case, the range of the quotient tested was from 0.28 to 1.70. The arithmetic mean and median in this case were much smaller than 1: 0.89 and 0.81, respectively. By calculating the K_L_ value from the linear forms, regardless of the equation used, it is possible to obtain values both smaller and larger than those calculated from the non-linear form.

The calculation of Langmuir isotherms using non-linear and linear forms is a topic that has been considered by other authors. Hamzaoui et al. 2018 analysed four different isotherms [54]. In two cases, the linear form IV gave the most similar qm results to those calculated from the non-linear form: in one case form II and in one case form III. Analysis of the K_L_ values revealed that in four cases, the most similar values to those calculated from the non-linear form were obtained when the linear IV form of the Langmuir isotherm was used and in one case when the linear II form was used. In these articles, conclusions were drawn on the basis of the analysis of few isotherms and mainly on the basis of evaluation by a single statistical measure (coefficient of determination). Similar observations to those presented in this article were made by Yadav and Singh (2017) during their study of fluoride sorption [55]. They found that the use of linear forms resulted in lower qm values and higher K_L_ values compared to the results obtained from non-linear forms. The most similar values of Langmuir constants to those calculated from the non-linear form were obtained when linear form II was used. Calculations of q_m_ and K_L_ from linear form I were the furthest from those obtained from the non-linear form. Similar relationships were also obtained, for example, by Subramanyam and Das 2014 and Tonk and Rápó 2022, and they obtained both smaller and larger values of q_m_ and K_L_ from the linear equations compared to those obtained from the non-linear equation [56,57]. Table 5 shows selected values of q_ml_/q_mn_ and K_LI_/K_Ln_ calculated from literature data.

The literature reports, as well as the results presented in this article, do not indicate a single linear form whose results are most similar to those obtained from the non-linear form of the Langmuir isotherm. Nevertheless, the most commonly used linear form II (Hanes–Woolf—often referred to as form I in the literature) is also the form that most often (but not always) gives the most similar qm and KL values to those calculated from the non-linear form. It should be noted that each of the proposed linearisations has certain limitations, as does the initial Langmuir isotherm [58].

Note that x (C_e_) and y (C_e_/q) are not independent. It follows that the correlation between C_e_ and Ce/q is overestimated. This can lead to a good fit of the results even when they are inconsistent with the Langmuir model. In contrast, the Lineweaver–Burke linearisation (I form), whose results deviate most from those obtained from the non-linear forms, is very sensitive to low values of q. The dependence of 1/q on 1/Ce leads to a clustering of points. Thus, the variability at low q values, i.e., high 1/q values, is particularly important. Any inaccuracy in the range of low equilibrium concentrations, and thus low capacities, affects the results very significantly. In the other linearisations (III Eadie-Hofstee and IV Scatchard), the x and y values are not independent and the correlation between x and y may be underestimated. For this reason, these equations may fit poorly even with data consistent with the Langmuir isotherm model.

### 3.2. Evaluation of Linear and Non-Linear Forms of the Langmuir Isotherm Based on the Coefficient of Determination R^2^

**Figure 3:**
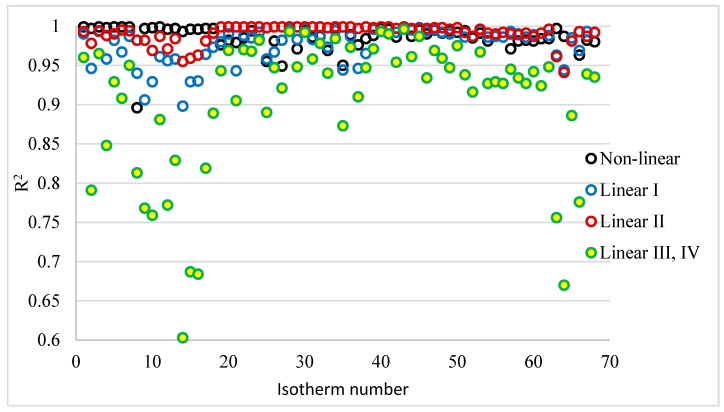
R^2^ values for the non-linear and linear form of Langmuir isotherms.

The coefficient of determination R^2^ is used to assess the fit of models to the test results obtained. It is usually the only statistical tool used for evaluating models of i.i.d. isotherms. In the supplement, the calculated Langmuir isotherms (non-linear and linear forms) with R^2^ coefficients of determination are shown in Tables S1–S11. For linear forms III and IV, the coefficients of determination are the same. This is because form III is a plot of the dependence of q on q/Ce, and form IV is the dependence of q/Ce on q. Detailed values of the coefficient of determination are presented in Figure 3.

Table 6 shows the ranges of the obtained R^2^ coefficients, arithmetic means, and medians. They show that the best fit was obtained when the II linear form of the Langmuir isotherm was used. The range of values, mean, and median for this linear form were the highest, and were clearly higher not only than the other linear forms, but also than the coefficients of determination of the non-linear form.

The highest coefficients of determination for the II linear form of the Langmuir isotherm were not obtained in every case. For each isotherm study, the forms were ranked according to the magnitude of R^2^. Five forms of isotherms were analysed: non-linear (N), linear I form (I), linear II form (II), linear III form (III), and linear IV form (IV). However, since the determination coefficients for form III and IV are the same, there are four places in Table 7. The first place was assigned to the form with the highest R^2^ value and the fourth place to the form for which the R^2^s were lowest. Table 7 summarises the number of isotherm forms occupying the corresponding first, second, third, and fourth places in the series. In order to assess the fit of the isotherms to the test results, the sum of the products of place in the series × number of isotherms (l × i) is given in Table 6. The smaller the value of the sum of the products in question, the higher the place in the series in question. From the analysis of the results in Table 7, it can be seen that the R^2^ value for the II form was 40 times the first place among the 68 isotherms analysed. For the non-linear form, R^2^ was in first place 21 times and in third place as many as 31 times. For the III and IV forms, the R^2^ coefficient for each isotherm was the lowest and ranked fourth in all cases analysed. Based on the sum of the products, the forms can be ranked as follows: II form > non-linear > I form > III = IV form.

From the data in Table 6 and Table 7, it can be seen that superimposing the linear II model on the Langmuir isotherm model allows a better fit of such a corrected isotherm to the test results obtained. This may be due to a mismatch between the Langmuir model and the test results presented in the paper. The model has many limitations, one of which is the assumption that the sorbent surface is homogeneous. In the studies presented here, commercial and modified activated carbons were considered, which had different surface chemistries. It is therefore possible that the superimposition of a linear model on the Langmuir isotherm model gives a better fit to the experimental results compared to the unmodified Langmuir model.

Higher values of the coefficients of determination for the linear form compared to the non-linear form have also been obtained by other researchers. In the case of Deb et al. 2023 [21] and Yadav and Singh 2017 [55], higher coefficients of determination were obtained for linear form I and II compared to the non-linear form. Hamzaoui et al. 2018 obtained higher coefficients of determination only for the II linear form compared to the non-linear form [54]. The other forms were characterised by lower R^2^ values. Different results were obtained by Tonk et al. 2022 [57]. They obtained the highest R^2^ coefficients when the non-linear form was used.

The most common isotherms used to describe sorption are the Langmuir and Freundlich isotherms. Among other things, the R^2^ factor is used to assess which of these isotherms better describes the test results obtained. Tables S1–S11 give the R^2^ coefficients for the Freundlich isotherms determined from the non-linear forms. Table 8 compares how many coefficients of determination are greater, equal, and less for the isotherms in question. Depending on the form of Langmuir isotherm determination used, however, different conclusions can be drawn. When the R^2^ determined from the non-linear equation of the Langmuir isotherm was compared, as many as 64 models had higher coefficients of determination than the Freundlich isotherm. When the Langmuir isotherm was calculated from the most popular linear form II, only 49 cases resulted in higher R^2^ compared to the Freundlich isotherm. For the rarely used linear forms III and IV, larger R^2^ coefficients for the Frondlich isotherm were obtained in 35 cases. The use of the non-linear form or linear forms of the Langmuir isotherms may also influence the choice of the isotherm model that best describes the sorption process. Similar results have also been obtained by other researchers [44,55].

### 3.3. Evaluation of Linear and Non-Linear Forms of Langmuir Isotherms Based on Different Statistical Tools

Most researchers assess the fit of the isotherms to the test results solely on the basis of the coefficient of determination R^2^. However, this coefficient has some limitations, e.g., it does not take into account the number of variables in the model. Increasing the number of variables always increases the R^2^, which can lead to more complex models having higher R^2^ values. The coefficient of determination tells us nothing about measurement errors or the distribution of the residuals. It is therefore important to treat the coefficient of determination R^2^ as an important (but not the only) indicator for model evaluation. In addition, the coefficient of determination was originally designed for linear regressions and does not always perform well when calculating non-linear equations. The same formula was used to calculate the R^2^ value regardless of whether linear or non-linear regression was analysed. In the case of linear regression, R^2^ determines what portion of the research is explained by the model, and the remainder is unexplained variance. For non-linear models, there is no longer such a clear statistical interpretation of the R^2^ value as in the case of linear regression. This is because the models do not have an analytical solution, so R^2^ is calculated after fitting the model based on predictions. The R^2^ value may be less intuitive in this case, and its interpretation is more difficult. Therefore, several statistical indices are increasingly being used in scientific articles to evaluate adsorption isotherm models [23,46,59,60,61].

Several statistical tools were used to evaluate the linear and non-linear models used, in addition to the coefficient of determination R^2^: average relative error (ARE), sum of squares of errors (SSEs), chi-squared statistics (λ^2^), sum of the absolute errors (SAEs), the hybrid fractional error function (HYBRID), and the root mean square error (RMSE). These are commonly used non-linear error functions [48].

The values of these indices for all isotherms are given in Supplementary Tables S1–S11. It should be emphasised that in the case of the fitting coefficient R^2^, the better the fit of the test results for a given isotherm, the closer the R^2^ value is to 1. In the case of the other indices, the fit is better when they reach lower values. The studies analysed in this paper relate to different sorbents; therefore, the concentrations and number of points used to determine the isotherms vary. It is therefore not possible to compare these indices for the entire pool of studies, but only to compare the results for individual isotherms. In The tables in the following chapters are limited to ranking isotherms, assuming that the isotherm with the lowest value of the analyzed indicator occupies the first place, and the one with the highest value of this indicator occupies the fifth place.The sum of all error values for the 68 isotherms analysed, the range of values they take, and the median are also given. This is additional (less important) information because, as mentioned earlier, the indices for isotherms performed under different conditions should not be directly compared. However, a comparison of the sets of results investigating the different ways of calculating the Langmuir isotherm constants (non-linear and four linear forms) is most appropriate.

#### 3.3.1. Average Relative Error (ARE)

The average relative error (ARE) is used to assess the accuracy of the model and is particularly useful for a wide range of data values. It aims to minimise the fractional error over the entire concentration range. When calculating the ARE, the quotients of the differences between the experimental data and those obtained from the analysed model and the experimental data should be calculated. The resulting values should be divided by the number of measurement points (with an average over all measurement points) and multiplied by 100 to obtain the result as a percentage. Since ARE can be applied to different scales and sizes of data, it can be used to describe studies with different ranges of [12,61]. This is the situation in the studies analysed because, for example, we are comparing the adsorption of different compounds from solutions of different concentrations. Tables S1–S11 show ARE results for the linear and non-linear forms of the Langmuir isotherm, and Table 9 shows the places in the series occupied by the different isotherm types. It was assumed that first place in the series denotes the isotherm form with the lowest ARE value (the best fit of the model to the test results), and fifth place is reserved for the highest value. Most often (in 32%), the first place in the series is occupied by the third linear form of the Langmuir isotherm. The last place (in 49%) is occupied by the first linear form of the Langmuir isotherm. The sum of the products of the number of isotherms and place in the series was also calculated, with the smallest value of this product obtained indicating the best fit of the test results to the isotherm form in question. On this basis, considering all 68 isotherms, these forms can be ranked as follows: III > IV > N > I > II. This ranking takes into account the comparison of isotherm forms among themselves, but does not analyse the value of the average relative error. Therefore, the sum of the ARE for all 68 isotherms analysed, the range of values, and the median were calculated. Considering the sum of the average relative error, the isotherm forms were ranked in a different order: N > III > IV > II > I. A slightly different ranking can be obtained for the median analysis: III > N > IV > II > I. It is therefore difficult to assess unequivocally, on the basis of ARE, the suitability of the forms for describing the research results. Linear form II, on the basis of R^2^, was assessed as best describing the research results. In this case, this form, depending on how ARE is interpreted, ranks either fourth or fifth in the series, i.e., next to form I, it describes the research results most poorly.

In studies by many authors analysing different isotherms, their evaluation based on R^2^ and ARE varies [13,54,62].

#### 3.3.2. Sum of the Absolute Errors (SAEs)

Sum of the absolute errors is the total absolute sum of the deviations of the values obtained from the model from the values obtained from the tests. It is, therefore, one way of measuring the accuracy of the model. A major drawback is that it provides a better fit at higher adsorbate concentrations [50,56,63].

SAE values for all isotherms are summarised in Tables S1–S11. Table 10 shows which place in the series the analysed forms of the Langmuir isotherms occupy based on SAE values. The smaller the SAE value, the better the fit of the isotherm model to the test results obtained. The best fit is the isotherm calculated from the non-linear form (40 isotherms ranked first in the series). This is also confirmed by the smallest values of the sum of errors for all isotherms, the range of values, and the median. The weakest fit on the basis of the analysed parameters was obtained when the Langmuir isotherm was calculated from the I non-linear form. In this case, the forms of the isotherms can be ranked according to the following series: N > II > III > IV > I. This ranking does not coincide with the ranks obtained from the analysis of R^2^ values. A different selection of isotherms based on R^2^ and based on SAE has also been observed by other researchers [48,64].

#### 3.3.3. Sum of Squares of Errors (SSEs)

Sum of squares of errors (SSEs) is used, particularly in linear regression, to assess the fit of models to actual results. It is the sum of squares of the differences between the predicted values and the actual values obtained during the tests. If the SSE takes on low values then the model is a good fit to the test results. SSE is, therefore, a similar measure to SAE, but squaring the differences means that large errors will affect the results more than small errors. The magnitude of the SSE will therefore depend, among other things, on the concentrations (for low concentrations, the differences will be smaller, and for higher concentrations these differences will be larger).

The SSE values for all 68 isotherms analysed, calculated from five forms of Langmuir models, are given in Tables S1–S11. Table 11 shows the places in the series (the first place in the series is obtained by the form of isotherm for which the smallest SSE values were obtained, and the last place in the series when the SSE is the largest). The following series was obtained in this case: N > II > III > IV > I. This series coincides with the ranking of these forms of isotherms based on the sum of the values of all errors, and on the median. This series overlaps with the series formed on the basis of the SAE discussed in Section 3.3.2. However, these are not the same results, because on the basis of SSE, the Langmuir isotherm calculated from the non-linear version for 61 isotherms was ranked 1, while on the basis of SAE it was ranked 1 for only 40 isotherms.

The evaluation of the forms from which the Langmuir isotherm coefficients were calculated on the basis of SAE does not coincide with the evaluation on the basis of R^2^. On the basis of the R^2^ value, linear form II was selected as the best descriptor of the results of the study. When assessing the fit of the isotherm form to the test results using SSE and SAE, the lowest results for these quantities were found when the non-linear form of the Langmuir isotherm was used. Other researchers also noted different results when they evaluated isotherms using R^2^ and SSE. Raoof and Nahid 2024 [59] and Qayoom et al. 2017 [63] obtained the same R^2^ values for the two isotherms, but quite different SSE values. Other researchers have also obtained a different ranking of isotherms based on R^2^ and SSE [48,50].

#### 3.3.4. Chi-Squared Statistics (λ^2^)

The non-linear chi-squared statistics measure (λ^2^) is one of the most commonly used measures for comparing different isotherms (when analysing articles whose authors considered other statistical measures, not just R^2^). It is calculated as the sum of the squares of the differences between the experimental data and the values calculated from the model and divided by the value predicted by the model. The smaller the difference between the actual value and the one obtained from the model, the smaller the λ^2^ value and the better the fit of the model to the experimental data.

The values of λ^2^ have been placed in Tables S1–S11 and Table 12 shows the places in the ranks created from the values of λ^2^. It was assumed, similarly to the previous statistical measures, that the first place in the row would be obtained by the form of the isotherm for which the smallest values of λ^2^ were obtained, and the last place in the row was obtained when λ^2^ was the largest. The following series was obtained in this case: III > N > IV > II > I. Such a series, however, does not take into account the value of this measure, but only compares these measures between different forms of calculating the coefficients of the Langmuir equation. The series looks slightly different when we add up the values of λ^2^ for the 68 isotherms analysed: N > III > II > IV > I. However, regardless of whether we analyse the place in the series on the basis of the number of isotherms or the value of the sum of λ^2^, linear form I is the least descriptive of the actual results.

The evaluation of the forms from which the Langmuir isotherm coefficients were calculated based on λ^2^ does not coincide with the evaluation based on R^2^. Similar observations were also noted by other researchers [29,50,54].

#### 3.3.5. Root Mean Square Error (RMSE)

The root mean square error (RMSE) is related to the SSE error (RMSE = √(SSE/n)). The RMSE measures the average error of the model fit, with the measure taking into account the number of measurement points. In addition, the unit of RMSE is the same as the data, which allows for easier interpretation of the results obtained compared to SSE. It is a more versatile measure because it takes into account the number of data, which makes it possible to compare different models that differ in the number of measurements. However, as errors are squared, larger errors, occurring at the higher concentrations used in the study, have the greatest impact on the magnitude of the RMSE. Several larger errors can significantly increase the RMSE even when the others have small values.

The RMSE values are shown in Tables S1–S11, and Table 13 shows the places in the series created based on the RMSE. Due to the definition of the RMSE and SSE error, the places in the series defined for these measures are the same. The series that can be created from the place in series is the same as the series for SSE: N > II > III > IV > I. A similar series occurs when analysing the values of the sum of all RMSEs for the 68 isotherms. The values of SSE and RMSE differ significantly, but the ranks are similar. This is mainly due to the fact that the same number of measurement points (8 points) were used to determine most of the analysed isotherms (50), and for 18 isotherms, 7 points were used. Analysing the sum of the RMSEs for the 68 isotherms resulted in the same series.

As with the errors analysed previously, the assessment of the best form of the Langmuir isotherm based on RMSE does not coincide with that based on R^2^. Similar observations have been presented in other articles [13,65].

#### 3.3.6. The Hybrid Fractional Error Function (HYBRID)

The previously discussed SSE/RMSE measures are inaccurate in certain cases (low concentrations). The hybrid fractional error function (HYBRID) was developed especially for low values of q_e_ (such values occur at low concentrations). It takes into account not only the number of degrees of freedom (n), but also the number of parameters (*p*). It is, therefore, especially relevant when comparing models with different numbers of parameters [66]. The HYBRID is expressed in %, so it is easy to compare values from different data sets. However, for very small q_e,exp_, HYBRID can take on very large values.

The HYBRID values are included in Tables S1–S11, and Table 14 shows the places in the series created from the HYBRID values. Based on the sum of the ixl products, a series was formed, according to which the best-fitting form of the Langmuir isotherm is linear form III and the worst-fitting is linear form I (III > N > IV > II > I). However, taking the sum of all HYBRID values for the 68 isotherms analysed, a slightly different series was obtained: N > III > IV > II > I. In this case, the calculation of the Langmuir isotherm parameters from the non-linear form turned out to be closest to the actual values.

#### 3.3.7. Comparison of Langmuir Isotherm Forms Based on Different Statistical Measures

Table 15 summarises the series describing the fit of the different forms (non-linear and four linear) from which the Langmuir isotherm coefficients were calculated. Two ranking criteria were used. The first is the ranked position in the series created for each set of measurement points for the isotherm. First place means for R^2^ the value closest to 1 and for the other errors the lowest values. First place thus means the best fit of the real data to the model on the basis of the statistical measures in question. The second criterion is the sum of the errors for the 68 isotherms considered. In the case of R^2^, the best fit of the model occurs when this sum is the largest, and for the other errors analysed when their sum is the smallest. Taking these two criteria into account for some errors (R^2^; ARE; λ^2^; HYBRID) we have slightly different series. Taking into account the sum of the errors for all the static measures analysed, the best fit of the Langmuir model to the test results was found when it was calculated from the non-linear form. However, taking the order criterion, it was found that for most of the isotherms, calculating them from the Hanes–Woolf model allows larger R^2^ values to be obtained than for those calculated from the non-linear model. Analysing this criterion for the statistical measure ARE, λ^2^ and HYBRID, it was found that the linear form of the Edie-Hofstee isotherm had lower errors than the model calculated from the non-linear form. In other cases, the non-linear form proved to be the best.

When comparing linear forms with each other, it is not possible to unambiguously designate one of them as best describing the actual results. It is, however, characteristic that the linear form I (Lineweaver–Burk) describes the results most poorly for almost all statistical measures. The exception is the coefficient of determination, which is most often used to evaluate adsorption isotherms. In the articles, the Hanes–Woolf linear model is used most often, but the Lineweaver–Burk model comes second, which coincides with the series created for R^2^. However, based on the other statistical measures, it can be concluded that using the linear Lineweaver–Burk form results in the largest errors.

Subramanyam and Das [56], in their analysis of six different errors, also found that the linear I form (Lineweaver–Burk) has the weakest fit to real data. The non-linear form of the Langmuir isotherm best describes the results of the study. For the three error types, the II linear form (Hanes–Woolf) came first: the form (Scatchard) in two cases and the III form (Edie-Hofstee) in one case.

## 4. Conclusions

Analysing literature reports and based on my own experience as a reviewer, I found that one can still find, in many scientific articles, the calculation of Langmuir isotherm coefficients from linear forms. In addition, many of the researchers compare sorbents in their articles by means of the maximum monolayer capacity calculated from the Langmuir isotherm, without specifying how the coefficients of this isotherm were calculated. Often, different isotherm models are also compared on the basis of one another. In the case of the Langmuir isotherm, the linear forms do not result from a mathematical transformation of the equation (as in the case of the Freundlich isotherm). Several different non-linear forms have been proposed, and their application gives different results. A second problem is the restriction of the error analysis to the coefficient of determination R^2^, which was originally designed for linear regression and does not always perform well for non-linear regression.

After analysing the 68 isotherms, it was found that, depending on how the isotherms were calculated (i.e., the non-linear and the four linear forms analysed), different values of the monolayer capacity q_m_ were obtained. For each of the linear models analysed, both smaller and larger values of q_m_ were obtained compared to the q_m_ calculated from the non-linear form of the isotherm. For linear models I, III and IV, the value of q_ml_/q_mn_ is mostly less than 1, and for the II linear form it is greater than 1. The range of q_ml_/q_mn_ values is closest to 1 for the II linear form (0.93–1.1). The q_m_ values calculated from this linear form are closest to those calculated from the non-linear form. By analysing the literature, it can be seen that this form (Hanes–Woolf) is the most commonly used for isotherm calculations using linear forms. The largest differences between q_m_ values calculated from the non-linear form and from the linear form occur when the linear form I (Lineweaver–Burk) is used for calculations. It should be noted that the Linear Lineweaver–Burk form is most sensitive to low concentrations because the 1/C value is large and sensitive to even small errors. Many of the isotherms points in the presented in this article are characterised by low concentrations. This can cause large errors in linear regression analysis and affect the accuracy of qm and K_L_ calculations. When collecting data on q_m_ values, it is advantageous to provide the method used to calculate the Langmuir isotherm. Such compilations should therefore only be regarded as indicative for sorbents differing significantly in their monolayer capacity.

When comparing Langmuir and Freundlich isotherms calculated from non-linear forms between each other, a significantly better fit to the test results was obtained for the Langmuir isotherm (in 64 cases out of 68 based on R^2^). When linear forms were used, the dominance of the Langmuir isotherm was not so great. The way the isotherms are calculated, in some cases, therefore influences the choice of the model that best describes the results of the study.

The assessment of the fit of the isotherm to the experimental data in most articles is based solely on the coefficient of determination R^2^. This coefficient has some limitations and was intended for linear regressions. Particularly now that most calculations are based on a non-linear form, however, one should consider extending the number of statistical measures. The analysis of statistical measures such as ARE, SAE, SSE, λ^2^, RMSE, and HYBRID concluded that the non-linear model best describes the experimental results. However, for the most commonly used coefficient of determination R^2^, the linear form II (Hanes–Woolf) proved to be the best. Of the linear models, depending on the method adopted and the error analysed, the best fit to the actual data was obtained with model III (Edie-Hofstee), which is, however, very rarely used to calculate Langmuir isotherm coefficients. Model I proved to be the model that offered the weakest description of reality.

The practical conclusions are as follows:

1. Due to the limitations of the coefficient of determination, different statistical measures should be used simultaneously to assess and compare isotherms.
2. It is necessary to calculate Langmuir isotherm coefficients from the non-linear form. It would be beneficial to introduce such guidelines in the information for authors of articles.
3. If a linear method must be used, Hanes–Woolf best preserves qm, whereas Eadie-Hofstee minimises the absolute error.
4. When comparing qm and KL values in an article with previously published studies, it would be beneficial to state how these values were calculated in the cited articles.
5. Drawing conclusions from a comparison of one’s own research results with early published studies is correct only if the method used to calculate the isotherms is the same or if the qm values differ significantly from one another (at least twice).
6. When comparing the fit of different isotherms calculated from non-linear forms to the obtained test results, do not rely only on the coefficient of determination, but use other statistical measures, e.g., SAE, λ2, RMSE.

## Figures and Tables

**Table 1:** Conditions of sorption measurements used to determine adsorption isotherms.

Adsorbate	Adsorbent	Initial Concentration, mg/L	Adsorbent Dose, g/L	Conditions Analysed	Number of Isotherms	Ref.
Oxytetracycline	7 commercial AC 5 modified AC	60–200	0.4	- pH	18	[39]
Sulfacetamide	7 commercial AC	20–300	4	- pH - temperature	20	[40]
Chromium(III) and nickel(II)	3 commercial AC	10–100	0.4	-	6	[41]
Ampicillin	6 commercial AC	40–200	4	- pH - temperature	18	[42]
Lead(II) and cadmium(II)	3 commercial AC	10–100	0.4	-	6	[43]

**Table 2:** Statistical measures used in the study.

Error	Equation	Best Fit	Ref.
Coefficient of determination (R^2^), -	$R^{2}=\frac{\sum_{n+1}^{n}{\left(q_{e.exp}-\bar{q_{e,cal}}\right)}^{2}}{\sum_{i=1}^{n}{\left(q_{e,exp}-\bar{q_{e,exp}}\right)}^{2}+\sum_{n+1}^{n}{\left(q_{e,exp}-q_{e,cal}\right)}^{2}}$	Lor close to 1	[12,47,48]
Average relative error (ARE), %	$ARE=\frac{100}{n}\sum_{i=1}^{n}\left|\frac{q_{e,exp}-q_{e,cal}}{q_{e,exp}}\right|$	Smallest value	[12,47,48]
Sum of squares of errors (SSE), mg/g	$SSE=\sum_{i=1}^{n}{\left(q_{e,cal}-q_{e,exp}\right)}^{2}$		[12,48]
Chi-squared statistics (λ^2^), mg/g	$\lambda^{2}=\frac{100}{n}\sum_{i=1}^{n}\frac{{\left(q_{e,exp}-q_{e,cal}\right)}^{2}}{q_{e,cal}}$		[47,49,50]
Sum of the absolute errors (SAE), mg/g	$SAE=\sum_{i=1}^{n}\left|q_{e,cal}-q_{e,exp}\right|$		[48,50]
The hybrid fractional error function (HYBRID), mg/g	$HYBRID=\frac{100}{n-p}\sum_{i=1}^{n}\frac{{\left(q_{e,cal}-q_{e,exp}\right)}^{2}}{q_{e,exp}}$		[12,49]
Root mean square error (RMSE), mg/g	$RMSE={\left(\frac{1}{n}\sum_{i=1}^{n}{\left(q_{e,exp}-q_{e,cal}\right)}^{2}\right)}^{\frac{1}{2}}$		[12,49]

n—number of measurement points; q_e,exp_—adsorption capacity resulting from measurements; q_e,cal_—adsorption capacity calculated using a particular model; *p*—number of isotherm parameters.

**Table 3:** The quotient of monolayer capacitance values calculated from the linear forms versus the non-linear form of the Langmuir isotherm (q_ml_/q_mn_, -). Statistical measures used in the study.

Form of Isotherm	Range q_mI_/q_mn_, -	Arithmetic Mean	Median	Number of Isotherms (Percentage of Isotherms, %)		
				q_mI_/q_mn_ < 1	q_mI_/q_mn_ = 1	q_mI_/q_mn_ > 1
I	0.83–1.75	1.09	1.08	46 (67.6)	2 (2.9)	20 (29.5)
II	0.93–1.1	1.00	0.99	19 (28.0)	9 (13.2)	40 (58.8)
III	0.90–1.09	1.00	1.01	38 (55.9)	4 (5.9)	26 (38.2)
IV	0.92–1.38	1.06	1.05	45 (66.2)	5 (7.4)	18 (26.4)

**Table 4:** The quotient of the value of the K_L_ constant from the linear form versus the non-linear form of the Langmuir isotherm (K_LI_/K_Ln_, -).

Form of Isotherm	Range K_LI_/K_Ln_, -	Arithmetic Mean	Median	Number of Isotherms (Percentage of Isotherms, %)		
				K_LI_/K_Ln_, <1	K_LI_/K_Ln_, =1	K_LI_/K_Ln_, >1
I	0.28–1.7	0.89	0.81	20 (29.4)	1 (1.5)	47 (69.1)
II	0.69–1.33	1.01	1.03	40 (58.8)	6 (8.8)	22 (32.4)
III	0.72–1.45	1.01	0.97	26 (38.2)	7 (10.3)	35 (51.5)
IV	0.51–1.36	0.92	0.88	20 (29.4)	1 (1.5)	47 (69.1)

**Table 5:** Values of q_ml_/q_mn_ for 68 isotherms and various linear forms based on information from scientific articles.

Range of Values: q_ml_/q_mn_	Range of Values: K_Ll_/K_Ln_	Ref.
0.54–0.94	1.5–16.3	[24]
0.59–0.73	10.9–16.3	[55]
0.97–1.13	0.06–1.66	[45]
0.87–1.03	0.82–1.63	
0.62–0.97	1.36–27.41	
0.94–1.03	0.16–1.55	
0.8–1.02	1.02–1.58	[56]

**Table 6:** R^2^ determination coefficients for non-linear and linear forms of Langmuir isotherms.

Forms of Isotherm	Range R^2^	Mean	Median	Number of Isotherms, -, (Percentage of Samples, %)				
				R^2^ ≥ 0.99	0.99 > R^2^ ≥ 0.98	0.98 > R^2^ ≥ 0.95	0.95 > R^2^ ≥ 0.9	R^2^ < 0.9
N	0.896–0.999	0.986	0.989	33 (48.5)	24 (35.3)	9 (13.2)	1 (1.5)	1 (1.5)
I	0.898–0.999	0.976	0.986	25 (36.8)	19 (27.9)	13 (19.1)	10 (14.7)	1 (1.5)
II	0.947–0.999	0.990	0.996	48 (70.6)	11 (16.2)	8 (1.7)	1 (1.5)	0 (0)
III i IV	0.603–0.996	0.907	0.939	5 (7.4)	3 (4.4)	16 (23.5)	25 (36.8)	19 (27.9)

**Table 7:** Ranking of models in terms of R^2^.

Form of Isotherm	Place in Line Due to R^2^ (i)				∑l × i	∑R^2^
	1	2	3	4		
	Number of Isotherms (l)					
N	21	16	31	0	146	67.044
I	7	28	33	0	162	66.366
II	40	24	4	0	100	67.352
III i IV	0	0	0	68	272	61.668

**Table 8:** Quantitative comparison of R^2^ determination coefficients for different forms of Langmuir isotherm and the non-linear form of the Freundlich isotherm.

Langmuir Isotherm Form	Number of Isotherms		
	R^2^(L) > R^2^(F)	R^2^(L) = R^2^(F)	R^2^(L) < R^2^(F)
N	64	0	4
I	47	0	21
II	49	1	18
III i IV	33	0	35

**Table 9:** Evaluation of average relative error ARE) for non-linear and linear forms of Langmuir isotherms.

Form of Isotherm	Place in Line Due to ARE (i)					∑l × i	∑ARE	Range ARE	Median
	1	2	3	4	5				
	Number of Isotherms (l)								
N	14	18	11	24	1	184	273	1.039–8.482	3.821
I	11	8	7	9	33	249	298	1.116–11.702	4.144
II	8	8	10	14	28	250	292	1.315–12.401	4.168
III	22	16	19	8	3	158	275	1.130–8.868	3.793
IV	13	18	21	13	3	179	277	1.133–9.029	3.899

**Table 10:** Evaluation of the sum of the absolute errors (SAEs) for the non-linear and linear forms of the Langmuir isotherms.

Form of Isotherm	Place in Line Due to SAE (i)					∑l × i	∑SAE	Range SAE	Median
	1	2	3	4	5				
	Number of Isotherms (l)								
N	40	14	9	4	1	116	679.988	2.353–26.455	8.781
I	2	2	3	10	51	310	976.381	2.953–58.306	11.856
II	14	25	12	7	10	178	716.861	2.729–30.406	8.597
III	6	20	25	15	2	191	779.079	28.24–4.775	9.356
IV	6	7	19	32	4	225	804.791	2.806–40.324	9.698

**Table 11:** Evaluation of sum of squares of errors (SSEs) for non-linear and linear forms of Langmuir isotherms.

Form of Isotherm	Place in Line Due to SSE (i)					∑l × i	∑SSE	Range SSE	Median
	1	2	3	4	5				
	Number of Isotherms (l)								
N	61	2	3	2	0	82	153.,443	0.745–130.272	13.823
I	0	0	1	9	58	329	4796.676	1.702–749.375	29.438
II	5	39	17	2	5	167	1735.761	1.574–150.794	14.84
III	1	17	33	16	1	203	2226.488	1.316–371.255	15.779
IV	1	10	14	39	4	239	2501.191	1.266–285.27	20.935

**Table 12:** Evaluation of chi-squared statistics (λ^2^) for non-linear and linear forms of Langmuir isotherms.

Form of Isotherm	Place in Line Due to λ^2^ (i)					∑l × i	∑ λ^2^	Range λ^2^	Median
	1	2	3	4	5				
	Number of Isotherms (l)								
N	14	35	15	3	1	146	48.086	0.026–3.714	0.427
I	0	0	3	18	47	316	82.865	0.048–6.645	0.779
II	3	17	16	18	14	227	58.837	0.052–6.106	0.538
III	35	12	15	5	1	129	51.722	0.044–3.698	0.446
IV	16	4	19	24	5	202	65.345	0.044–10.574	0.478

**Table 13:** Evaluation of root mean square error (RMSE) for non-linear and linear forms of Langmuir isotherms.

Form of Isotherm	Place in Line Due to RMSE (i)					∑l × i	∑RMSE	Range RMSE	Median
	1	2	3	4	5				
	Number of Isotherms (l)								
N	61	2	3	2	0	82	101.34	0.305–4.035	1.366
I	0	0	1	9	58	329	163.73	0.461–9.678	1.894
II	5	39	17	2	5	167	106.52	0.443–4.342	1.362
III	1	17	33	16	1	203	117.03	0.406–6.812	1.449
IV	1	10	14	39	4	239	124.40	0.398–5.971	1.618

**Table 14:** Evaluation The hybrid fractional error function (HYBRID) for non-linear and linear forms of Langmuir isotherms.

Form of Isotherm	Place in Line Due to HYBRID (i)					∑l ∗ i	∑HYBRID	Range HYBRID	Median
	1	2	3	4	5				
	Number of Isotherms (l)								
N	9	39	18	2	0	149	819.557	0.443–50.802	7.498
I	0	0	2	19	47	317	1419.721	0.793–102.696	13.537
II	9	15	9	20	15	221	942.310	0.854–84.587	9.141
III	37	8	16	5	2	131	871.297	0.729–60.705	7.618
IV	13	6	23	22	4	202	940.763	0.721–58.847	8.446

**Table 15:** Ranking of models based on error magnitude.

Error	Ranking Criterion	
	Order	Sum of Errors
R^2^	II > N > I > III = IV	II > N > I > III = IV
ARE	III > IV > N > I > II	N > III > IV > II > I
SAE	N > II > III > IV > I	N > II > III > IV > I
SSE	N > II > III > IV > I	N > II > III > IV > I
λ^2^	III > N > IV > II > I	N > III > II > IV > I
RMSE	N > II > III > IV > I	N > II > III > IV > I
HYBRID	III > N > IV > II > I	N > III > IV > II > I

## Data Availability

The original contributions presented in this study are included in the article/Supplementary Materials. Further inquiries can be directed to the author.

## Supplementary Materials

The following supporting information can be downloaded at: https://www.mdpi.com/article/10.3390/ma18153506/s1, Table S1: Oxytetracycline adsorption isotherms on different activated carbons. Langmuir model in non-linear and linear forms; Table S2: Isotherms of oxytetracycline adsorption from solutions with different pH values. Langmuir mod-el in non-linear and linear forms; Table S3: Oxytetracycline adsorption isotherms on modified activated carbons. Langmuir model in non-linear and linear forms; Table S4: Isotherms of sulfacetamide adsorption on different activated carbons. Langmuir model in non-linear and linear forms; Table S5: Isotherms of sulfacetamide adsorption from solutions at different pH values. Langmuir model in non-linear and linear forms; Table S6: Isotherms of sulfacetamide adsorption from solutions at different temperatures. Langmuir model in non-linear and linear forms; Table S7: Adsorption isotherms of Cr(III) and Ni(II) on different activated carbons. Langmuir model in non-linear and linear forms; Table S8: Adsorption isotherms of Pb(II) and Cd(II) on different activated carbons. Langmuir model in non-linear and linear forms; Table S9: Isotherms of adsorption of ampicillins on different activated carbons. Langmuir model in non-linear and linear forms; Table S10: Isotherms of adsorption of ampicillins from solutions with different pH. Langmuir model in non-linear and linear forms; Table S11: Isotherms of adsorption of ampicillins from solutions of different tempers. Langmuir model in non-linear and linear forms.
