# Supplementary material for: Analysis of the Differences Resulting from the Determination of Langmuir Isotherm Coefficients from Linear and Non-Linear Forms—A Case Study
Source: Materials (Basel). 2025 Jul 26;18(15):3506. doi: 10.3390/ma18153506 (PMC12347600; doi:10.3390/ma18153506)
Supplement: Supplementary file 1 [file materials-18-03506-s001.zip › materials-3724064-supplementary.docx]

Supplement

Analysis of the differences resulting from the determination of Langmuir isotherm coefficients from linear and non-linear forms - a case study

Joanna Lach^1^*

**Table S1.** Oxytetracycline adsorption isotherms on different activated carbons. Langmuir model in non-linear and linear forms.

| **Constants of the isotherm model** | **Non-linear form** | **Linear form I** | **Linear form II** | **Linear form III** | **Linear form IV** |
| --- | --- | --- | --- | --- | --- |
| ROW 08 pH6 | | | | | |
| q_m_, mg/g  K_L_, L/mg  q_mI_/q_mn_  K_LI_/K_Ln_  R^2^  ARE  SSE  λ^2^  RMSE  HYBRID  SAE | 55.13^a^  0.028^a^  -  -  0.999^a^; 0.997*^a^  2.161  5.389  0.166  0.821  2.839  5.370 | 58.48  0.024  1.06  0.86  0.990  1.942  7.892  0.204  0.993  3.527  5.138 | 54.35  0.029  0.99  1.04  0.993  2.244  5.564  0.179  0.834  3.040  5.530 | 56.24  0.026  1.02  0.93  0.960  2.073  5.717  0.163  0.845  2.765  5.316 | 54.14  0.025  0.98  0.89  0.960  2.057  6.367  0.173  0.892  2.959  5.366 |
| WG-12 pH6 | | | | | |
| q_m_, mg/g  K_L_, L/mg  q_mI_/q_mn_  K_LI_/K_Ln_  R^2^  ARE  SSE  λ2  RMSE  HYBRID  SAE | 65.94^a^  0.033^a^  -  -  0.997^a^; 0.993*^a^  4,794  28.487  0.924  1.887  16.748  12.065 | 79.39  0.022  1.20  0.67  0.946  5.674  64.617  1.480  2.842  25.557  17.279 | 64.94  0.034  0.98  1.03  0.978  4.922  29.622  0.956  1.924  16.905  12.689 | 66.68  0.032  1.01  0.97  0.791  4.679  29.152  0.914  1.909  15.953  12.235 | 74.15  0.025  1.12  0.76  0.791  5.026  43.595  1.112  2.334  18.802  14.472 |
| F-300 pH6 | | | | | |
| q_m_, mg/g  K_L_, L/mg  q_mI_/q_mn_  K_LI_/K_Ln_  R^2^  ARE  SSE  λ^2^  RMSE  HYBRID  SAE | 73.31^a^  0.043^a^  -  -  0.999^a^; 0.996*^a^  2.397  11.336  0.258  1.190  4.373  7.999 | 77.52  0.037  1.06  0.86  0.993  2.152  16.401  0.389  1.432  5.328  7.755 | 71.94  0.045  0.98  1.05  0.994  2.597  12.022  0.289  1.226  4.859  8.483 | 74.92  0.040  1.02  0.93  0.965  2.311  12.216  0.252  1.236  4.251  8.024 | 76.14  0.039  1,04  0.91  0.965  2.234  13.732  0.269  1.310  4.592  7.892 |
| F-100 pH6 | | | | | |
| q_m_, mg/g  K_L_, L/mg  q_mI_/q_mn_  K_LI_/K_Ln_  R^2^  ARE  SSE  λ^2^  RMSE  HYBRID  SAE | 37.45^a^  0.030^a^  -  -  0.998^a^; 0.996*^a^  3.579  7.977  0.361  0.999  6.112  6.412 | 40.98  0.023  1.09  0.77  0.958  3.799  11.822  0.450  1.216  7.445  7.695 | 36.50  0.032  0.97  1.07  0.988  3.620  8.355  0.391  1.022  6.658  6.299 | 38.06  0.029  1.02  0.97  0.848  3.447  8.161  0.356  1.010  5.886  6.382 | 40.39  0.024  1.08  0.8  0.848  3.640  10.743  0.418  1.159  6.880  7.274 |
| WACC 8x30 pH6 | | | | | |
| q_m_, mg/g  K_L_, L/mg  q_mI_/q_mn_  K_LI_/K_Ln_,  R^2^  ARE  SSE  λ^2^  RMSE  HYBRID  SAE | 44.35^a^  0.026^a^  -  -  0.999^a^; 0.997*^a^  2.481  5.522  0.212  0.831  3.644  5.073 | 47.62  0.022  1.07  0.85  0.982  2.706  8.079  0.259  1.005  4.461  5.985 | 43.48  0.028  0.98  1.08  0.990  2.591  5.745  0.231  0.847  3.973  5.245 | 45.34  0.025  1.02  0.96  0,929  2.539  5.818  0,207  0.853  3.524  5.329 | 46.77  0.023  1.05  0.88  0.929  2.586  6.971  0.230  0.933  3.942  5.596 |
| WAZ 0.6-2.4 pH6 | | | | | |
| q_m_, mg/g  K_L_, L/mg  q_mI_/q_mn_  K_LI_/K_Ln_  R^2^  ARE  SSE  λ^2^  RMSE  HYBRID  SAE | 29.87^a^  0.031^a^  -  -  0.999^a^; 0.998*^a^  2.281  2.605  0.147  0.571  2.480  3.356 | 31.55  0.026  1.06  0.84  0.967  2.816  3.589  0.177  0.670  2.893  4.570 | 29.50  0.032  0.99  1.03  0.995  2.154  2.661  0.154  0.577  2.605  3.071 | 30.19  0.029  1.01  0.94  0.908  2.377  2.657  0.146  0.576  2.413  3.585 | 31.12  0.027  1.04  0,87  0.908  2.671  3.188  0.163  0.631  2.645  4.253 |
| Picabiol pH6 | | | | | |
| q_m_, mg/g  K_L_, L/mg  q_mI_/q_mn_  K_LI_/K_Ln_  R^2^  ARE  SSE  λ^2^  RMSE  HYBRID  SAE | 59.79^a^  0.033^a^  -  -  0.999^a^; 0.997*^a^  2.188  6.796  0.218  0.922  3.757  5.540 | 64.52  0.027  1.08  0.82  0.987  2.598  12.478  0.304  1.249  5.214  7.393 | 59.17  0.34  0.99  1.03  0.994  2.258  6.933  0.228  0.931  3.934  5.698 | 61.13  0.031  1.02  0.94  0.950  2.320  7.372  0.213  0.960  3.599  6.125 | 62.50  0.029  1.05  0.88  0.950  2.400  8.785  0.234  1.048  3.961  6.528 |
| ^*^ Determination coefficient R^2^ for the linear version of the Freundlich isotherm  ^a^ Data provided in the article by Lach et al. 2021 | | | | | |

Table S2. Isotherms of oxytetracycline adsorption from solutions with different pH values. Langmuir model in non-linear and linear forms.

| **Constants of the isotherm model** | **Non-linear form** | **Linear form I** | **Linear form II** | **Linear form III** | **Linear form IV** |
| --- | --- | --- | --- | --- | --- |
| WG pH3 T=20^o^C | | | | | |
| q_m_, mg/g  K_L_, L/mg  q_mI_/q_mn_  K_LI_/K_Ln_  R^2^  ARE  SSE  λ^2^  RMSE  HYBRID  SAE | 56.44^a^  0.030^a^  -  -  0.896^a^; 0.995*^a^  4.644  17.959  0.713  1.498  12.924  9.554 | 64.10  0.022  1.14  0.73  0.940  5.1068  31.745  0.926  1.992  15.487  13.225 | 55.86  0.031  0.99  1.03  0.982  4.123  18.950  0.687  1.539  12.079  9.218 | 56.49  0.030  1.00  1.00  0.813  4.205  18.800  0.675  1.533  11.783  9.553 | 61.76  0.024  1.09  0.8  0.813  4.839  25.378  0.784  1.781  13.139  12.153 |
| WG pH10 T=20^o^C | | | | | |
| q_m_, mg/g  K_L_, L/mg  q_mI_/q_mn_  K_LI_/K_Ln_  R^2^  ARE  SSE  λ^2^  RMSE  HYBRID  SAE | 46.29^a^  0.036^a^  -  -  0.997^a^; 0.994*^a^  4.250  18.605  0.674  1.525  12.025  9.622 | 51.02  0.027  1.10  0.75  0.906  4.891  25.814  0.894  1.796  14.929  10.906 | 45.05  0.039  0.97  1.08  0.982  4.829  18.673  0.753  1.528  13.808  9.915 | 45.92  0.036  0.99  1.00  0.768  4.791  18.110  0.721  1.505  12.952  9.949 | 50.69  0.028  1.10  0.78  0.768  4.799  24.753  0.857  1.759  14.493  10.592 |
| ROW pH3 T=20^o^C | | | | | |
| q_m_, mg/g  K_L_, L/mg  q_mI_/q_mn_  K_LI_/K_Ln_  R^2^  ARE  SSE  λ^2^  RMSE  HYBRID  SAE | 50.66^a^  0.022^a^  -  -  0.998^a^; 0.995*^a^  4.490  15.138  0.651  1.376  11.760  8.524 | 59.77  0,015  1.18  0.68  0.929  4.995  25.485  0.885  1.785  14.790  10.752 | 50.51  0.022  1.00  1.00  0.969  4.523  15.314  0.655  1.384  11.609  8.710 | 50.13  0.022  0.99  1.00  0.759  4.558  15.378  0.661  1.386  11.786  8.749 | 56.97  0.017  1.12  0.77  0.759  4.656  21.266  0.769  1.630  12.961  9.659 |
| ROW pH10 T=20^o^C | | | | | |
| q_m_, mg/g  K_L_, L/mg  q_mI_/q_mn_  K_LI_/K_Ln_  R^2^  ARE  SSE  λ^2^  RMSE  HYBRID  SAE | 42.72^a^  0.024^a^  -  -  0.999^a^; 0.997*^a^  3.289  6.338  0.286  0.890  4.958  6.008 | 46.30  0.020  1.08  0.83  0.961  3.063  9.022  0.350  1.062  5.843  6.006 | 42.19  0.025  0.99  1.04  0.987  3.364  6.423  0.294  0.896  5.104  6.137 | 43.04  0.024  1.01  1.00  0.881  3.261  6.402  0.283  0.895  4.839  6.041 | 45.33  0.021  1.06  0.88  0.881  3.013  7.838  0.315  0.990  5.269  5.756 |
| F-300 pH3 T=20^o^C | | | | | |
| q_m_, mg/g  K_L_, L/mg  q_mI_/q_mn_  K_LI_/K_Ln_  R^2^  ARE  SSE  λ^2^  RMSE  HYBRID  SAE | 69.04^a^  0.038^a^  -  -  0.996^a^; 0.990*^a^  5.425  49.439  1.317  2.486  23.090  15.749 | 83.33  0.025  1.21  0.66  0.956  5.794  96.087  1.891  3.466  33.821  19.729 | 66.67  0.041  0.97  1.08  0.971  5.735  51.944  1.428  2.548  24.691  16.614 | 64.94  0.035  0.94  0.92  0.772  7.843  141.68  3.698  4.208  53.293  26.128 | 79.73  0.027  1.15  0.71  0.772  5.430  77.663  1.605  3.116  28.058  18.048 |
| F-300 pH10 T=20^o^C | | | | | |
| q_m_, mg/g  K_L_, L/mg  q_mI_/q_mn_  K_LI_/K_Ln_  R^2^  ARE  SSE  λ^2^  RMSE  HYBRID  SAE | 63.59^a^  0.039^a^  -  -  0.997^a^; 0.993*^a^  4.561  28.161  0.850  1.876  15.001  12.135 | 73.53  0.028  1.16  0.72  0.958  4.984  55.869  1.252  2.643  21.539  15.627 | 62.11  0.042  0.98  1.08  0.984  4.804  29.251  0.903  1.912  15.875  12.790 | 64.94  0.037  1.02  0.95  0.829  4.327  29.234  0.831  1.911  14.141  12.056 | 70.35  0.031  1.11  0.79  0.829  4.454  42.075  1.001  2.293  17.085  13.370 |
| ^*^ Determination coefficient R^2^ for the linear version of the Freundlich isotherm  ^a^ Data provided in the article by Lach et al. 2021 | | | | | |

**Table S3.** Oxytetracycline adsorption isotherms on modified activated carbons. Langmuir model in non-linear and linear forms.

| **Constants of the isotherm model** | **Non-linear form** | **Linear form I** | **Linear form II** | **Linear form III** | **Linear form IV** |
| --- | --- | --- | --- | --- | --- |
| WG-s | | | | | |
| q_m_, mg/g  K_L_, L/mg  q_mI_/q_mn_  K_LI_/K_Ln_  R^2^  ARE  SSE  λ^2^  RMSE  HYBRID  SAE | 74.66^a^  0.040^a^  -  -  0.993^a^; 0.988*^a^  7.586  77.831  2.271  3.119  44.157  20.375 | 100.00  0.021  1.34  0.53  0,898  8.427  183.599  3.695  4.791  64.426  28.729 | 74.07  0.039  0.99  0.95  0.955  7.573  80.725  2.297  3.177  41.157  21.614 | 72.89  0.041  0.98  1.03  0.603  7.719  80.687  2.331  3.176  42.970  21.562 | 92.92  0.025  1.24  0.63  0.603  7.760  138.181  2.974  4.156  51.298  25.561 |
| WG-CD | | | | | |
| q_m_, mg/g  K_L_, L/mg  q_mI_/q_mn_  K_LI_/K_Ln_  R^2^  ARE  SSE  λ^2^  RMSE  HYBRID  SAE | 84.74^a^  0.038^a^  -  -  0.996^a^; 0.991*^a^  6.716  64.690  1.769  2.844  32.740  19.735 | 112.36  0,022  1.33  0.58  0.929  6.878  172.729  3.110  4.647  55.178  25.055 | 84.03  0.037  0.99  1.03  0.959  6.758  67.377  1.809  2.902  31.968  20.583 | 83.54  0.040  0.99  1.05  0.687  6.801  66.877  1.813  2.891  32.430  20.494 | 101.09  0.026  1.19  0.68  0.687  5.991  108.908  2.233  3.690  38.281  20.435 |
| WGE-CD | | | | | |
| q_m_, mg/g  K_L_, L/mg  q_mI_/q_mn_  K_LI_/K_Ln_  R^2^  ARE  SSE  λ^2^  RMSE  HYBRID  SAE | 88.69^a^  0.041^a^  0.996^a^; 0.991*^a^  6.479  67.433  1.857  2.903  33.974  18.840 | 117.65  0.0237  1.33  0.58  0.930  7.229  185.971  3.338  4.821  57.272  27.900 | 89.29  0.039  1.01  0.95  0.963  6.42  70.796  1.883  2.975  32.203  19.772 | 86,87  0.042  0.98  1.02  0.684  6.615  69.413  1.911  2.946  34.238  19.553 | 99.74  0.036  1.12  0.88  0.684  8.394  145.566  3.026  4.266  58.847  27.284 |
| WGE-AIR | | | | | |
| q_m_, mg/g  K_L_, L/mg  q_mI_/q_mn_  K_LI_/K_Ln_  R^2^  ARE  SSE  λ^2^  RMSE  HYBRID  SAE | 89.65^a^  0.046^a^  -  -  0.997^a^; 0.993*^a^  5.076  43.896  1.151  2.342  19.865  15.568 | 106.38  0.0321  1.23  0.70  0.964  5.316  94.832  1.763  3.443  29.248  20.904 | 89.29  0.0449  1.00  0.98  0.981  5.093  45.744  1.171  2.391  19.443  16.225 | 90.32  0.044  1.01  0.96  0.819  5.01  45.096  1.146  2.374  19.023  16.000 | 105.72  0.029  1.18  0.66  0.819  6.452  125.563  3.078  3.962  44.595  23.870 |
| WG-AIR | | | | | |
| q_m_, mg/g  K_L_, L/mg  q_mI_/q_mn_  K_LI_/K_Ln_  R^2^  ARE  SSE  λ^2^  RMSE  HYBRID  SAE | 89.50^a^  0.057^a^  -  -  0.997^a^: 0.993*^a^  5.238  49.392  1.233  2.485  21.456  16.823 | 102.04  0.044  1.14  0.77  0.973  4.459  83.656  1.405  3.234  24.062  18.431 | 86.96  0.061  0.97  1.07  0.991  4.212  31.256  0.813  1.977  14.0.27  13.537 | 89.95  0.057  1.01  1.00  0.889  3.915  31.834  0.769  1.995  12.948  13.070 | 94.99  0.050  1.06  0.88  0.889  3.955  44.76  0.903  2.365  14.979  14.844 |
| ^*^ Determination coefficient R^2^ for the linear version of the Freundlich isotherm  ^a^ Data provided in the article by Lach et al. 2021 | | | | | |

**Table S4.** Isotherms of sulfacetamide adsorption on different activated carbons. Langmuir model in non-linear and linear forms.

| **Constants of the isotherm model** | **Non-linear form** | **Linear form I** | **Linear form II** | **Linear form III** | **Linear form IV** |
| --- | --- | --- | --- | --- | --- |
| ROW 08 sulfacetamide pH6 | | | | | |
| q_m_, mg/g  K_L_, L/mg  q_mI_/q_mn_  K_LI_/K_Ln_  R^2^  ARE  SSE  λ^2^  RMSE  HYBRID  SAE | 54.95^b^  0.395^b^  -  -  0.976^b^; 0.964*^b^  5.096  28.887  0.967  1.948  15.037  13.037 | 51.02  0.530  0.92  1.34  0.980  4.822  61.422  1.347  2.771  21.115  15.967 | 57.80  0.313  1.04  0.79  0.999  7.229  48.010  2.320  2.450  31.949  16.537 | 52.87  0.476  0.95  1.20  0.943  4.648  40.120  0.940  2.239  15.401  14.421 | 53.77  0.449  0.97  1.13  0.943  4.834  34.080  0.857  2.064  14.178  14.138 |
| WG-12 sulfacetamide pH6 | | | | | |
| q_m_, mg/g  K_L_, L/mg  q_mI_/q_mn_, -  K_LI_/K_Ln_, -  R^2^  ARE  SSE  λ^2^  RMSE  HYBRID  SAE | 48.97^b^  0.133^b^  -  -  0.982^b^; 0.999*^b^  3.417  12.893  0.373  1.269  6.259  9.038 | 47.17  0.153  0.96  1.15  0.990  3.114  17.867  0.435  1.494  7.097  9.402 | 50.76  0.115  1.04  0.86  0.999  4.470  18.029  0.693  1.501  11.014  9.951 | 47.98  0.145  0.98  1.09  0.969  3.164  14.655  0.369  1.353  6.190  9.158 | 48.44  0.141  0.99  1.06  0.969  3.187  13.622  0.355  1.305  6.012  8.986 |
| F-300 sulfacetamide pH6 | | | | | |
| q_m_, mg/g  K_L_, L/mg  q_mI_/q_mn_  K_LI_/K_Ln_  R^2^  ARE  SSE  λ^2^  RMSE  HYBRID  SAE | 53.01^b^  0.352^b^  -  -  0.979^b^ ; 0.927*^b^  4.828  22.105  1.100  1.662  15.622  10.876 | 47.85  0.424  0.90  1.20  0.943  6.695  84.426  2.321  3.249  34.055  20.111 | 54.35  0.312  1.03  0.89  0.999  4.167  25.742  1.569  1.794  20.261  8.421 | 50.26  0.448  0.95  1.27  0.905  6.228  41.079  1.185  2.266  19.835  16.597 | 51.63  0.406  0.97  1.15  0.905  5.704  28.887  1.001  1.900  16.227  14.158 |
| F-100 sulfacetamide | | | | | |
| q_m_, mg/g  K_L_, L/mg  q_mI_/q_mn_  K_LI_/K_Ln_  R^2^  ARE  SSE  λ^2^  RMSE  HYBRID  SAE | 39.79^b^  0.111^b^  -  -  0.985^b^; 0.961*^b^  2.813  5.424  0.209  0.823  3.456  5.985 | 38.61  0.127  0.97  1.14  0.988  2.508  7.938  0.245  0.996  4.076  6.217 | 40.82  0.097  1.03  0.87  0.999  3.369  7.762  0.391  0.985  6.032  6.462 | 39.12  0.121  0.98  1.09  0.970  2.512  6.372  0.207  0.892  3.493  5.856 | 39.41  0.117  0.99  1.05  0.970  2.623  5.824  0.198  0.853  3.348  5.925 |
| WACC 8x30 sulfacetamide | | | | | |
| q_m_, mg/g  K_L_, L/mg  q_mI_/q_mn_  K_LI_/K_Ln_  R^2^  ARE  SSE  λ^2^  RMSE  HYBRID  SAE | 48.89^b^  0.037^b^  -  -  0.994^b^; 0.954*^b^  1.995  2.936  0.140  0.606  2.367  3.631 | 50.76  0.034  1.04  0.92  0.985  2.409  4.640  0.183  0.762  2.979  4.968 | 49.02  0.037  1.00  1.00  0.999  2.005  2.945  0.140  0.607  2.346  3.670 | 49.04  0.037  1.00  1.00  0,968  1.993  2.963  0.140  0.609  2.336  3.645 | 49.64  0.036  1.02  0.97  0.968  2.059  3.232  0.146  0.636  2.399  3.878 |
| WAZ 0,6-2,4 sulfacetamide | | | | | |
| q_m_, mg/g  K_L_, L/mg  q_mI_/q_mn_  K_LI_/K_Ln_  R^2^  ARE  SSE  λ^2^  RMSE  HYBRID  SAE | 34.58^b^  0.099^b^  -  -  0.983^b^; 0.945*^b^  1.870  2.632  0.095  0.574  1.610  3.944 | 34.13  0.102  0.99  1.03  0.992  1.916  2.943  0.102  0.606  1.691  4.215 | 35.34  0.085  1.02  0.86  0.999  2.220  3.310  0.162  0.643  2.595  4.082 | 34.37  0.100  0.99  1.01  0.982  1.808  2.553  0.091  0.565  1.520  3.874 | 34.52  0.098  1.00  0.99  0.982  1.744  2.419  0.088  0.550  1.483  3.668 |
| ^*^ Determination coefficient R^2^ for the linear version of the Freundlich isotherm  ^b^ Data provided in the article by Lach 2022 | | | | | |

**Table S5.** Isotherms of sulfacetamide adsorption from solutions at different pH values. Langmuir model in non-linear and linear forms.

| **Constants of the isotherm model** | **Non-linear form** | **Linear form I** | **Linear form II** | **Linear form III** | **Linear form IV** |
| --- | --- | --- | --- | --- | --- |
| WG pH6 T=30^o^C | | | | | |
| q_m_, mg/g  K_L_, L/mg  q_mI_/q_mn_  K_LI_/K_Ln_  R^2^  ARE  SSE  λ^2^  RMSE  HYBRID  SAE | 47.57^b^  0.104^b^  -  -  0.969^b^; 0.949*^b^  4.652  20.291  0.755  1.593  11.865  11.230 | 42.92  0.147  0.90  1.41  0.973  5.026  50.642  1.315  2.516  20.040  14.649 | 46.08  0.113  0.97  1.09  0.998  4.338  23.222  0.730  1.704  11.500  11.269 | 44.17  0.135  0.93  1.30  0.940  4.787  36.162  0.953  2.126  14.973  13.310 | 44.93  0.127  0.94  1.22  0.940  4.694  29.631  0.807  1.925  12.830  12.1723 |
| WG pH6 T=40^o^C | | | | | |
| q_m_, mg/g  K_L_, L/mg  q_mI_/q_mn_  K_LI_/K_Ln_  R^2^  ARE  SSE  λ^2^  RMSE  HYBRID  SAE | 41.86^b^  0.093^b^  -  -  0.998^b^; 0.914*^b^  1.039  0.745  0.026  0.305  0.443  2.353 | 42.37  0.084  1.01  0.90  0,994  1.879  2.888  0.116  0.601  1.866  4.005 | 41.32  0.093  0.99  1.00  0.999  1.368  2.074  0.068  0.509  1.113  3.390 | 42.16  0.085  1.01  0.91  0.984  1.807  2.765  0.109  0.588  1.763  3.873 | 42.37  0.084  1.01  0.90  0.984  1.930  3.031  0.122  0.616  1.964  4.102 |
| ROW pH6 T=30^o^C | | | | | |
| q_m_, mg/g  K_L_, L/mg  q_mI_/q_mn_  K_LI_/K_Ln_  R^2^  ARE  SSE  λ^2^  RMSE  HYBRID  SAE | 52.48^b^  0.302^b^  -  -  0.950^b^; 0.961*^b^  7.865  52.029  2.165  2.550  31.917  18.720 | 46.95  0.489  0.89  1.62  0.944  7.609  114.736  2.758  3.787  44.018  23.842 | 55.25  0.238  1.05  0.79  0.999  8.651  67.399  3.949  2.903  49.275  18.339 | 49.14  0.424  0.94  1.40  0.873  7,072  79,226  2,012  3.147  34.267  20.544 | 50.92  0.370  0.97  1.23  0.873  7.271  61.988  1.779  2.784  30.466  19.604 |
| ROW pH6 T=40^o^C | | | | | |
| q_m_, mg/g  K_L_, L/mg  q_mI_/q_mn_  K_LI_/K_Ln_  R^2^  ARE  SSE  λ^2^  RMSE  HYBRID  SAE | 41.49^b^  0.114^b^  -  -  0.989^b^; 0.935*^b^  2.422  4.469  0.184  0.747  3.037  3.037 | 40.10  0.130  0.97  1.14  0.987  2.852  7.651  0.240  0.978  4.019  7.007 | 41.84  0.109  1.01  0.96  0.999  2.569  4.773  0.220  0.772  3.530  5.092 | 40.81  0.123  0.98  1.08  0.973  2.423  5.319  0.184  0.815  3.122  5.551 | 41.13  0.120  0,99  1,05  0.973  2.320  4.843  0.176  0.778  2.996  5.156 |
| F-300 pH6 T=30^o^C | | | | | |
| q_m_, mg/g  K_L_, L/mg  q_mI_/q_mn_  K_LI_/K_Ln_  R^2^  ARE  SSE  λ^2^  RMSE  HYBRID  SAE | 48.59^b^  0.269^b^  -  -  0.976^b^; 0.927*^b^  4.518  19.056  0.945  1.543  13.606  9.941 | 44.64  0.384  ‘0.92  1.43  0.946  6.160  53.344  1.440  2.582  23.551  17.098 | 49.75  0.241  1.02  0.90  0.997  4.031  22.137  1.289  1.663  17.239  8.090 | 46.44  0.339  0.96  1.26  0.910  5.665  31.395  0.961  1.981  16.205  14.401 | 47,53  0.308  0.98  1.14  0.910  5.235  23.297  0.836  1.707  13.655  12.486 |
| F-300 pH6 T=40^o^C | | | | | |
| q_m_, mg/g  K_L_, L/mg  q_mI_/q_mn_  K_LI_/K_Ln_  R^2^  ARE  SSE  λ^2^  RMSE  HYBRID  SAE | 42.40^b^  0.221^b^  -  -  0.984^b^; 0.911*^b^  3.045  7.689  0.375  0.980  5.825  6.149 | 40.65  0.272  0.96  1.23  0.965  3.904  15.524  0.498  1.393  8.493  9.504 | 42.92  0.208  1.01  0.94  0.999  2.830  8.374  0.452  1.023  6.724  5.337 | 41.42  0.252  0.98  1.14  0.947  3.636  10.591  0.381  1.151  6.500  8.315 | 41.92  0.239  0.99  1.08  0.947  3.415  8.691  0.350  1.042  5.832  7.426 |
| ^*^ Determination coefficient R^2^ for the linear version of the Freundlich isotherm  ^b^ Data provided in the article by Lach 2022 | | | | | |

**Table S6.** Isotherms of sulfacetamide adsorption from solutions at different temperatures. Langmuir model in non-linear and linear forms.

| **Constants of the isotherm model** | **Non-linear form** | **Linear form I** | **Linear form II** | **Linear form III** | **Linear form IV** |
| --- | --- | --- | --- | --- | --- |
| WG pH6 T=30^o^C | | | | | |
| q_m_, mg/g  K_L_, L/mg  q_mI_/q_mn_  K_LI_/K_Ln_  R^2^  ARE  SSE  λ^2^  RMSE  HYBRID  SAE | 47.57^b^  0.104^b^  -  -  0.969^b^; 0.949*^b^  4.652  20.291  0.755  1.593  11.865  11.230 | 42.92  0.147  0.90  1.41  0.973  5.026  50.642  1.315  2.516  20.040  14.649 | 46.08  0.113  0.97  1.09  0.998  4.338  23.222  0.730  1.704  11.500  11.269 | 44.17  0.135  0.93  1.30  0.940  4.787  36.162  0.953  2.126  14.973  13.310 | 44.93  0.127  0.94  1.22  0.940  4.694  29.631  0.807  1.925  12.830  12.1723 |
| WG pH6 T=40^o^C | | | | | |
| q_m_, mg/g  K_L_, L/mg  q_mI_/q_mn_  K_LI_/K_Ln_  R^2^  ARE  SSE  λ^2^  RMSE  HYBRID  SAE | 41.86^b^  0.093^b^  -  -  0.998^b^; 0.914*^b^  1.039  0.745  0.026  0.305  0.443  2.353 | 42.37  0.084  1.01  0.90  0,994  1.879  2.888  0.116  0.601  1.866  4.005 | 41.32  0.093  0.99  1.00  0.999  1.368  2.074  0.068  0.509  1.113  3.390 | 42.16  0.085  1.01  0.91  0.984  1.807  2.765  0.109  0.588  1.763  3.873 | 42.37  0.084  1.01  0.90  0.984  1.930  3.031  0.122  0.616  1.964  4.102 |
| ROW pH6 T=30^o^C | | | | | |
| q_m_, mg/g  K_L_, L/mg  q_mI_/q_mn_  K_LI_/K_Ln_  R^2^  ARE  SSE  λ^2^  RMSE  HYBRID  SAE | 52.48^b^  0.302^b^  -  -  0.950^b^; 0.961*^b^  7.865  52.029  2.165  2.550  31.917  18.720 | 46.95  0.489  0.89  1.62  0.944  7.609  114.736  2.758  3.787  44.018  23.842 | 55.25  0.238  1.05  0.79  0.999  8.651  67.399  3.949  2.903  49.275  18.339 | 49.14  0.424  0.94  1.40  0.873  7,072  79,226  2,012  3.147  34.267  20.544 | 50.92  0.370  0.97  1.23  0.873  7.271  61.988  1.779  2.784  30.466  19.604 |
| ROW pH6 T=40^o^C | | | | | |
| q_m_, mg/g  K_L_, L/mg  q_mI_/q_mn_  K_LI_/K_Ln_  R^2^  ARE  SSE  λ^2^  RMSE  HYBRID  SAE | 41.49^b^  0.114^b^  -  -  0.989^b^; 0.935*^b^  2.422  4.469  0.184  0.747  3.037  3.037 | 40.10  0.130  0.97  1.14  0.987  2.852  7.651  0.240  0.978  4.019  7.007 | 41.84  0.109  1.01  0.96  0.999  2.569  4.773  0.220  0.772  3.530  5.092 | 40.81  0.123  0.98  1.08  0.973  2.423  5.319  0.184  0.815  3.122  5.551 | 41.13  0.120  0,99  1,05  0.973  2.320  4.843  0.176  0.778  2.996  5.156 |
| F-300 pH6 T=30^o^C | | | | | |
| q_m_, mg/g  K_L_, L/mg  q_mI_/q_mn_  K_LI_/K_Ln_  R^2^  ARE  SSE  λ^2^  RMSE  HYBRID  SAE | 48.59^b^  0.269^b^  -  -  0.976^b^; 0.927*^b^  4.518  19.056  0.945  1.543  13.606  9.941 | 44.64  0.384  ‘0.92  1.43  0.946  6.160  53.344  1.440  2.582  23.551  17.098 | 49.75  0.241  1.02  0.90  0.997  4.031  22.137  1.289  1.663  17.239  8.090 | 46.44  0.339  0.96  1.26  0.910  5.665  31.395  0.961  1.981  16.205  14.401 | 47,53  0.308  0.98  1.14  0.910  5.235  23.297  0.836  1.707  13.655  12.486 |
| F-300 pH6 T=40^o^C | | | | | |
| q_m_, mg/g  K_L_, L/mg  q_mI_/q_mn_  K_LI_/K_Ln_  R^2^  ARE  SSE  λ^2^  RMSE  HYBRID  SAE | 42.40^b^  0.221^b^  -  -  0.984^b^; 0.911*^b^  3.045  7.689  0.375  0.980  5.825  6.149 | 40.65  0.272  0.96  1.23  0.965  3.904  15.524  0.498  1.393  8.493  9.504 | 42.92  0.208  1.01  0.94  0.999  2.830  8.374  0.452  1.023  6.724  5.337 | 41.42  0.252  0.98  1.14  0.947  3.636  10.591  0.381  1.151  6.500  8.315 | 41.92  0.239  0.99  1.08  0.947  3.415  8.691  0.350  1.042  5.832  7.426 |
| ^*^ Determination coefficient R^2^ for the linear version of the Freundlich isotherm  ^b^ Data provided in the article by Lach 2022 | | | | | |

**Table S7.** . Adsorption isotherms of Cr(III) and Ni(II) on different activated carbons. Langmuir model in non-linear and linear forms.

| **Constants of the isotherm model** | **Non-linear form** | **Linear form I** | **Linear form II** | **Linear form III** | **Linear form IV** |
| --- | --- | --- | --- | --- | --- |
| ROW 08 Cr(III) | | | | | |
| q_m_, mg/g  K_L_, L/mg  q_mI_/q_mn_  K_LI_/K_Ln_  R^2^  ARE  SSE  λ^2^  RMSE  HYBRID  SAE | 63.49^c^  0.129^c^  -  -  0.988^c^; 0.863*^c^  3.444  16.362  0.427  1.430  7.429  10.140 | 67.11  0.105  1.06  0.81  0.996  3.175  31.71  0.580  1.991  10.091  12.160 | 61.35  0.147  0.97  1.14  0.998  4.391  22.574  0.727  1.680  13.217  11.292 | 65.22  0.115  1.03  0.89  0.971  3.126  20.808  0.413  1.613  7.009  11.015 | 65.87  0.111  1.04  0.86  0.971  3.132  23.685  0.451  1.721  7.724  11.368 |
| WG-12 Cr(III) | | | | | |
| q_m_, mg/g  K_L_, L/mg  q_mI_/q_mn_  K_LI_/K_Ln_  R^2^  ARE  SSE  λ^2^  RMSE  HYBRID  SAE | 60.73^c^  0.080^c^  -  -  0.997^c^; 0.929*^c^  1.423  2.984  0.081  0.611  1.351  4.160 | 60.98  0.078  1.00  0.96  0,999  1.364  3.198  0.080  0.632  1.319  4.273 | 60.24  0.082  0.99  1.02  0.999  1.612  3.163  0.094  0.629  1.588  4.288 | 61.19  0.078  1.01  0.96  0.993  1.331  3.165  0.078  0.629  1.296  4.212 | 61.34  0.077  1.01  0.96  0.993  1.369  3.271  0.079  0.639  1.317  4.307 |
| F-300 Cr(III) | | | | | |
| q_m_, mg/g  K_L_, L/mg  q_mI_/q_mn_  K_LI_/K_Ln_  R^2^  ARE  SSE  λ^2^  RMSE  HYBRID  SAE | 55.85^c^  0.106^c^  -  -  0.996^c^; 0.907*^c^  1.980  3.393  0.116  0.651  1.955  4.811 | 57.47  0.095  1.03  0.90  0.999  1.662  6.125  0.145  0.875  2.397  5.613 | 55.25  0.109  0.99  1.03  0.999  2.059  3.787  0.141  0.688  2.410  4.590 | 56.74  0.099  1.02  0.93  0.990  1.736  4.359  0.112  0.738  1.845  5.179 | 56.96  0.098  1.02  0.92  0.990  1.26  4.743  0.118  0.770  1.946  5.318 |
| ROW Ni(II) | | | | | |
| q_m_, mg/g  K_L_, L/mg  q_mI_/q_mn_  K_LI_/K_Ln_  R^2^  ARE  SSE  λ^2^  RMSE  HYBRID  SAE | 47.48^c^  0.104^c^  -  -  0.986^c^; 0.869*^c^  3.438  8.910  0.365  1.055  6.583  6.924 | 51.28  0.080  1.08  0.77  0.992  3.807  22.823  0.592  1.689  10.210  10.439 | 46.08  0.116  0.97  1.12  0.997  4.349  11.171  0.516  1.182  9.626  8.422 | 48.96  0.092  1.03  0.88  0.954  3.425  11.732  0.356  1.211  6.028  8.176 | 49.69  0.088  1.05  0.85  0.954  3.473  14.350  0.405  1.339  6.864  8.700 |
| WG Ni(II) | | | | | |
| q_m_, mg/g  K_L_, L/mg  q_mI_/q_mn_  K_LI_/K_Ln_  R^2^  ARE  SSE  λ^2^  RMSE  HYBRID  SAE | 51.70^c^  0.067^c^  -  -  0.998^c^; 0.960*^c^  1.151  1.485  0.045  0.431  0.737  2.895 | 51.02  0.070  0.99  1.04  0.999  1.116  1.782  0.048  0.472  0.793  3.115 | 52.08  0.066  1.01  0.99  0.999  1.315  1.574  0.052  0.443  0.854  3.091 | 51.33  0.069  0.99  1.03  0,996  1.130  1.577  0.044  0.444  0.729  3.056 | 51.39  0.069  0.99  1.03  0.996  1.133  1.549  0.044  0.440  0.721  3.030 |
| F-300 Ni(II) | | | | | |
| q_m_, mg/g  K_L_, L/mg  q_mI_/q_mn_  K_LI_/K_Ln_  R^2^  ARE  SSE  λ^2^  RMSE  HYBRID  SAE | 40.29^c^  0.082^c^  -  -  0.987^c^; 0.888*^c^  3.580  5.876  0.271  0.857  4.649  6.290 | 42.92  0.067  1.07  0.82  0.995  3.022  11.219  0.359  1.184  6.018  7.293 | 39.53  0.087  0.98  1.06  0.997  3.851  6.399  0.324  0.894  5.673  6.348 | 41.50  0.073  1.03  0.89  0.961  3.091  7.290  0.263  0.954  4.313  6.467 | 42.11  0.070  1.05  0.85  0.961  3.063  8.632  0.291  1.039  4.821  6.824 |
| * Determination coefficient R^2^ for the linear version of the Freundlich isotherm  ^c^ Data provided in the article by Lach and Okoniewska 2023 | | | | | |

**Table S8.** Adsorption isotherms of Pb(II) and Cd(II) on different activated carbons. Langmuir model in non-linear and linear forms.

| **Constants of the isotherm model** | **Non-linear form** | **Linear form I** | **Linear form II** | **Linear form III** | **Linear form IV** |
| --- | --- | --- | --- | --- | --- |
| ROW 08 Pb(II) | | | | | |
| q_m_, mg/g  K_L_, L/mg  q_mI_/q_mn_  K_LI_/K_Ln_  R^2^  ARE  SSE  λ^2^  RMSE  HYBRID  SAE | 135.94^d^  0.128^d^  -  -  0.995^d^; 0.916*^d^  2.438^d^  38.78^d^  0.445^d^  2.202  7.567^d^  13.461^d^ | 144,93  0.111  1.06  0.87  0.998  2.106  95.345  0.831  3.452  14.801  14.349 | 133.33  0.134  0.98  1.05  0.997  2.777  44.320  0.559  2.354  9.404  14.396 | 138.61  0.120  1.02  0.94  0.987  2.215  44.633  0.437  2.362  7.438  13.595 | 139.44  0.119  1.03  0.93  0.987  2.175  47.798  0.459  2.444  7.854  13.565 |
| WG-12 Pb(II) | | | | | |
| q_m_, mg/g  K_L_, L/mg  q_mI_/q_mn_  K_LI_/K_Ln_  R^2^  ARE  SSE  λ^2^  RMSE  HYBRID  SAE | 162.19^d^  0.167^d^  -  -  0.990^d^; 0.966*^d^  6.809^d^  130.272^d^  3.714^d^  4.035  50.802^d^  26.455^d^ | 135.14  0.289  0.83  1.70  0,994  6.937  749.375  6.645  9.678  102.696  58.306 | 161.29  0.181  0.99  1.06  0.997  6.864  147.004  3.049  4.287  45.794  30.165 | 145.30  0.247  0.90  1.45  0.934  6.282  371.255  3.582  6.812  60.705  44.775 | 149.29  0.231  0.92  1.36  0.934  6.303  285.27  3.002  5.971  51.835  40.324 |
| F-300 Pb(II) | | | | | |
| q_m_, mg/g  K_L_, L/mg  q_mI_/q_mn_  K_LI_/K_Ln_  R^2^  ARE  SSE  λ^2^  RMSE  HYBRID  SAE | 149.11^d^  0.155^d^  -  -  0.995^d^; 0.952*^d^  4.115^d^  47.835^d^  1.182^d^  2.445  17.556^d^  16.345^d^ | 131.58  0.218  0.88  1.41  0.996  4.902  310.549  2.886  6.230  45.162  37.697 | 149.25  0.161  1.00  1.04  0.998  4.244  60.781  1.047  2.756  16.789  20.137 | 139.94  0.191  0.94  1.23  0.969  4.093  119.697  1.263  3.868  21.070  26.189 | 141.73  0.185  0,95  1.19  0.969  3.865  97.912  1.102  3.498  18.500  23.455 |
| ROW Cd(II) | | | | | |
| q_m_, mg/g  K_L_, L/mg  q_mI_/q_mn_  K_LI_/K_Ln_  R^2^  ARE  SSE  λ^2^  RMSE  HYBRID  SAE | 69.91^d^  0.135^d^  -  -  0.991^d^; 0.871*^d^  3.483^d^  16.322^d^  0.512^d^  1.428^d^  9.404^d^  9.736^d^ | 76.34  0.102  1.09  0.76  0.992  4.261  61.083  1.054  2.763  18.337  16.973 | 68.49  0.145  0.98  1.07  0.998  3.793  18.946  0.666  1.539  12.631  9.558 | 71.96  0.119  1.03  0.88  0.959  3.742  22.893  0.501  1.692  8.484  12.598 | 72.94  0.114  1.04  0.84  0.959  3.770  28.428  10.574  1.885  9.685  13.258 |
| WG Cd(II) | | | | | |
| q_m_, mg/g  K_L_, L/mg  q_mI_/q_mn_  K_LI_/K_Ln_  R^2^  ARE  SSE  λ^2^  RMSE  HYBRID  SAE | 126.34^d^  0.123^d^  -  -  0.994^d^; 0.903*^d^  4.119^d^  43.699^d^  0.990^d^  2.337  19.060^d^  15.015^d^ | 151.51  0.082  1.20  0.67  0.990  6.338  448.638  4.103  7.489  76.039  40.685 | 125.00  0.124  0.99  1.01  0.996  4.169  47.711  1.018  2.442  19.154  16.246 | 131.40  0.107  1.04  0.87  0,947  4.306  67.781  0.973  2.911  16.900  19.451 | 137.70  0.102  1.09  0.83  0.947  4.530  95.309  1.185  3.452  20.486  22.271 |
| F-300 Cd(II) | | | | | |
| q_m_, mg/g  K_L_, L/mg  q_mI_/q_mn_  K_LI_/K_Ln_  R^2^  ARE  SSE  λ^2^  RMSE  HYBRID  SAE | 104.42^d^  0.131^d^  -  -  0.992^d^; 0.886*^d^  3.827^d^  36.218^d^  0.689^d^  2.128  12.064^d^  14.762^d^ | 113.64  0.104  1.09  0.79  0.998  3.376  109.021  1.189  3.692  21.140  20.654 | 102.04  0.139  0.98  1.06  0.998  4.542  42.199  0.884  2.297  15.618  17.197 | 108.22  0.115  1.04  0.88  0.975  3.018  52.301  0.666  2.557  11.247  15.898 | 109.31  0.113  1.05  0.86  0.975  2.973  60.097  0.732  2.741  12.467  16.454 |
| * Determination coefficient R^2^ for the linear version of the Freundlich isotherm  ^d^ Data provided in the article by Lach and Okoniewska 2024 | | | | | |

**Table S9.** Isotherms of adsorption of ampicillins on different activated carbons. Langmuir model in non-linear and linear forms.

| **Constants of the isotherm model** | **Non-linear form** | **Linear form I** | **Linear form II** | **Linear form III** | **Linear form IV** |
| --- | --- | --- | --- | --- | --- |
| ROW 08 ampicillin pH6 | | | | | |
| q_m_, mg/g  K_L_, L/mg  q_mI_/q_mn_  K_LI_/K_Ln_  R^2^  ARE  SSE  λ^2^  RMSE  HYBRID  SAE | 64.74^e^  0.027^e^  -  -  0.994^e^; 0.956*^e^  2.840  5.736  0.286  0.847  5.278  5.160 | 75.76  0.019  1.17  0.70  0.986  4.413  28.494  0.703  1.887  12.128  11.694 | 65.36  0.036  0.992  1.01  1.33  3.107  6.148  0.275  0.877  4.878  6.117 | 66.92  0.024  1.03  0.89  0.938  3.162  6.986  0.277  0.934  4.785  6.462 | 89.11  0.023  1.38  0.85  0.938  3.377  9.697  0.322  1.101  5.458  7.450 |
| WG-12 ampicillin pH6 | | | | | |
| q_m_, mg/g  K_L_, L/mg  q_mI_/q_mn_  K_LI_/K_Ln_  R^2^  ARE  SSE  λ^2^  RMSE  HYBRID  SAE | 82.34^e^  0.034^e^  -  -  0.985^e^; 0.938*^e^  4.542  25.203  0.689  1.775  11.459  13.017 | 101.01  0.024  1.23  0.71  0,987  5.691  97.848  1.665  3.497  30.407  20.000 | 81.30  0.036  0.99  1.06  0.987  4.385  19.513  0.603  1.562  10.647  11.426 | 84.99  0.033  1.03  0.97  0.916  3.976  23.212  0.597  1.703  10.286  11.149 | 89.09  0.030  1.08  0.88  0.916  4.269  33.516  0.723  2.047  12.472  12.818 |
| F-300 ampicillin pH6 | | | | | |
| q_m_, mg/g  K_L_, L/mg  q_mI_/q_mn_  K_LI_/K_Ln_  R^2^  ARE  SSE  λ^2^  RMSE  HYBRID  SAE | 65.72^e^  0.052^e^  -  -  0.991^e^; 0.924*^e^  3.335  10.738  0.339  1.159  5.901  8.269 | 71.94  0.041  1.09  0.79  0.995  3.130  28.882  0.569  1.900  9.936  10.805 | 64.94  0.053  0.99  1.02  0.996  3.549  11.211  0.365  1.184  6.325  8.852 | 67.98  0.047  1.03  0.90  0.967  2.973  13.737  0.328  1.310  5.526  8.601 | 69.01  0.045  1.05  0.87  0.967  2.924  16.348  0.364  1.430  6.168  8.922 |
| F-100 ampicillin | | | | | |
| q_m_, mg/g  K_L_, L/mg  q_mI_/q_mn_  K_LI_/K_Ln_  R^2^  ARE  SSE  λ^2^  RMSE  HYBRID  SAE | 49.54^e^  0.033^e^  -  -  0.981^e^; 0.910*^e^  3.919  10.974  0.406  1.171  6.991  8.126 | 55.56  0.025  1.12  0.76  0.986  4.520  27.294  0.735  1.847  13.057  11.001 | 46.85  0.036  0.95  1.09  0.991  4.367  11.181  0.469  1.182  8.271  8.475 | 50.74  0.031  1.02  0.94  0.927  3.930  12.267  0.412  1.238  7.058  8.576 | 52,45  0.028  1.06  0.85  0.927  4.143  15.863  0.478  1.408  8.247  9.463 |
| WACC 8x30 | | | | | |
| q_m_, mg/g  K_L_, L/mg  q_mI_/q_mn_  K_LI_/K_Ln_  R^2^  ARE  SSE  λ^2^  RMSE  HYBRID  SAE | 56.04^e^  0.030^e^  -  -  0.987^e^; 0.931*^e^  3.755  9.379  0.397  1.083  7.171  7.210 | 65.36  0.021  0.987  1.17  0.70  4.672  31.958  0.796  1.999  14.095  12.100 | 55.87  0.030  1.00  1.00  0.990  3.840  9.690  0.394  1.101  6.972  7.706 | 58.40  0.027  1.04  0.90  0,929  3.716  11.371  0.381  1.192  6.524  8.231 | 60.56  0.025  1.08  0.83  0.929  3.945  15.290  0.447  1.382  7.673  9.240 |
| WAZ 0,6-2,4 | | | | | |
| q_m_, mg/g  K_L_, L/mg  q_mI_/q_mn_  K_LI_/K_Ln_  R^2^  ARE  SSE  λ^2^  RMSE  HYBRID  SAE | 52.75^e^  0.032^e^  -  -  0.986^e^; 0.925*^e^  3.905  9.156  0.402  1.070  7.260  7.439 | 60.98  0.023  1.16  0.72  0.986  4.871  29.993  0.783  1.936  13.759  12.322 | 52.36  0.032  0.99  1.00  0.992  3.888  9.534  0.408  1.091  7.226  7.500 | 54.88  0.029  1.04  0.91  0.927  3.843  11.106  0.387  1.178  6.600  8.371 | 56.90  0.027  1.08  0.84  0.927  4.059  15.073  0.457  1.373  7.803  9.327 |
| * Determination coefficient R^2^ for the linear version of the Freundlich isotherm  ^e^ Data provided in the article by Lach 2024 | | | | | |

**Table S10.** Isotherms of adsorption of ampicillins from solutions with different pH. Langmuir model in non-linear and linear forms.

| **Constants of the isotherm model** | **Non-linear form** | **Linear form I** | **Linear form II** | **Linear form III** | **Linear form IV** |
| --- | --- | --- | --- | --- | --- |
| WG pH2 T=20^o^C | | | | | |
| q_m_, mg/g  K_L_, L/mg  q_mI_/q_mn_  K_LI_/K_Ln_  R^2^  ARE  SSE  λ^2^  RMSE  HYBRID  SAE | 40,32^e^  0.020^e^  -  -  0.971^e^; 0.923*^e^  3.590  8.321  0.344  1.020  5.623  6.862 | 41.67  0.020  1.03  1.00  0.993  3.213  9.156  0.326  1.70  5.711  6.323 | 37.31  0.026  0.93  1.30  0.990  3.732  5.205  0.259  0.807  4.419  5.822 | 39.32  0.023  0.98  1.15  0.945  3.366  5.743  0.233  0.847  3.944  5.941 | 40.39  0.021  0.945  1.00  1.05  3.261  6.933  0.261  0.931  4.483  6.073 |
| WG pH10 T=20^o^C | | | | | |
| q_m_, mg/g  K_L_, L/mg  q_mI_/q_mn_  K_LI_/K_Ln_  R^2^  ARE  SSE  λ^2^  RMSE  HYBRID  SAE | 58.70^e^  0.031^e^  -  -  0.981^e^; 0.917*^e^  3.815  15.604  0.465  1.397  7.806  9.460 | 65,79  0,025  1.21  0.81  0,990  4.281  36.723  0.832  2.143  15.032  12.017 | 56.18  0.035  0.96  1.13  0.989  4,370  14.366  0.511  1.340  8.877  9.748 | 59.79  0.030  1.02  0.97  0.934  3.544  16.179  0.455  1.422  7.798  8.760 | 61.75  0.028  1.05  0.90  0.934  3.750  20.604  0.525  1.605  9.116  9.736 |
| ROW pH2 T=20^o^C | | | | | |
| q_m_, mg/g  K_L_, L/mg  q_mI_/q_mn_  K_LI_/K_Ln_  R^2^  ARE  SSE  λ^2^  RMSE  HYBRID  SAE | 48.84^e^  0.035^e^  -  -  0.982^e^; 0.910*^e^  4.085  10.402  0.428  1.140  7.638  7.870 | 55.56  0.025  1.14  0.71  0.986  4.520  27.294  0.435  1.847  13.057  11.001 | 47.85  0.036  0.98  1.03  0.991  4.367  11.181  0.469  1.182  8.271  8.475 | 50.74  0.031  1.04  0.89  0.927  3.930  12.267  0.412  1.238  7.058  8.576 | 52.45  0.029  1.07  0.83  0.927  4.144  15.863  0.478  1.408  8.247  9.463 |
| ROW pH10 T=20^o^C | | | | | |
| q_m_, mg/g  K_L_, L/mg  q_mI_/q_mn_  K_LI_/K_Ln_  R^2^  ARE  SSE  λ^2^  RMSE  HYBRID  SAE | 47.25^e^  0.025^e^  -  -  0.981^e^; 0.921*^e^  4.145  8.851  0.369  1.052  6.396  7.494 | 53.763  0.019  1.14  0.76  0.989  3.967  20.810  0.610  1.613  10.823  9.032 | 46.296  0.026  0.98  1.04  0.987  4.440  9.052  0.398  1.064  6.887  7.972 | 48.28  0.028  1.02  1.12  0.942  4.873  11.729  0.589  1.211  11.092  7.525 | 49.50  0.027  1.05  1.08  0.942  4.382  9.704  0.470  1.101  8.644  7.061 |
| F-300 pH2 T=20^o^C | | | | | |
| q_m_, mg/g  K_L_, L/mg  q_mI_/q_mn_  K_LI_/K_Ln_  R^2^  ARE  SSE  λ^2^  RMSE  HYBRID  SAE | 46.44^e^  0.032^3^  0.984^e^; 0.917*^e^  3.576  8.064  0.332  1.004  5.803  6.7001 | 51.814  0.024  1.12  0.76  0.991  3.848  18.250  0.516  1.510  9.118  9.084 | 45.66  0.033  0,98  1,03  0.991  3.856  8.345  0.366  1.021  6.420  7.112 | 48.98  0.023  1.05  0.72  0,924  8.125  42.246  1.754  2.298  26.232  16.849 | 50.87  0.021  1.10  0.66  0.924  9.039  48.352  2.102  2.458  31.119  18.236 |
| F-300 pH10 T=20^o^C | | | | | |
| q_m_, mg/g  K_L_, L/mg  q_mI_/q_mn_  K_LI_/K_Ln_  R^2^  ARE  SSE  λ^2^  RMSE  HYBRID  SAE | 47.38^e^  0.053^e^  -  -  0.984^e^  0.894*^e^; 3.564  9.126  0.352  1.068  6.220  7.493 | 51.55  0.042  1.09  0.79  0.988  3.995  21.245  0.562  1.630  9.823  10.276 | 46.08  0.058  0.97  1.09  0.996  4.001  10.577  0.442  1.150  7.915  8.054 | 48.71  0.048  1.03  0.91  0.948  3.524  10.781  0.342  1.161  5.833  8.226 | 49.69  0.046  1.05  0.87  0.948  3.632  13.280  0.388  1.288  6.628  8.779 |
| * Determination coefficient R^2^ for the linear version of the Freundlich isotherm  ^e^ Data provided in the article by Lach 2024 | | | | | |

**Table S11.** Isotherms of adsorption of ampicillins from solutions of different tempers. Langmuir model in non-linear and linear forms.

| **Constants of the isotherm model** | **Non-linear form** | **Linear form I** | **Linear form II** | **Linear form III** | **Linear form IV** |
| --- | --- | --- | --- | --- | --- |
| WG pH6 T=30^o^C | | | | | |
| q_m_, mg/g  K_L_, L/mg  q_mI_/q_mn_  K_LI_/K_Ln_  R^2^  ARE  SSE  λ^2^  RMSE  HYBRID  SAE | 79.26^e^  0.032^e^  -  -  0.997^e^; 0.912*^e^  7.369  42.064  1.532  2.293  30.103  16.105 | 120.48  0.015  1.52  0.47  0.963  9.697  256.884  4.430  5.667  84.414  32.688 | 80.65  0.030  1.02  0.94  0.961  7.386  46.912  1.516  2.422  27.131  18.075 | 81.68  0.029  1.03  0.91  0.756  7.256  46.628  1.484  2.414  26.568  17.792 | 95.45  0.022  1.20  0.69  0.756  7.217  86.771  1.953  3.293  34.067  21.067 |
| WG pH6 T=40^o^C | | | | | |
| q_m_, mg/g  K_L_, L/mg  q_mI_/q_mn_  K_LI_/K_Ln_  R^2^  ARE  SSE  λ^2^  RMSE  HYBRID  SAE | 67.94^e^  0.039^e^  -  -  0.987^e^; 0.914*^e^  8.482  53.144  1.984  2.577  40.253  17.764 | 119.05  0.013  1.75  0.33  0,943  11.703  275.129  5.654  5.864  101.749  36.209 | 74.63  0.029  1.10  0.74  0.947  8.532  59.790  2.021  2.734  36.684  20.064 | 73.98  0.028  1.09  0.72  0.670  8.868  78.938  2.506  3.141  41.364  22.583 | 92.17  0.02  1.36  0.51  0.670  8.570  112.25  2.643  3.746  46.737  23.339 |
| ROW pH6 T=30^o^C | | | | | |
| q_m_, mg/g  K_L_, L/mg  q_mI_/q_mn_  K_LI_/K_Ln_  R^2^  ARE  SSE  λ^2^  RMSE  HYBRID  SAE | 58.90^e^  0.025^e^  -  -  0.981^e^; 0.891*^e^  5.124  14.752  0.612  1.358  11.126  9.809 | 73.53  0.016  1.25  0.76  0.985  5.522  52.790  1.258  2.569  22.813  14.748 | 58.82  0.025  1.00  1.19  0.981  5.247  15.871  0.612  1.408  10.628  10.775 | 62.01  0.022  1.05  1.05  0.886  4.692  17.438  0.582  1.476  9.944  10.142 | 66.15  0.020  1.12  0.95  0.886  4.596  25.428  0.711  1.783  12.280  11.095 |
| ROW pH6 T=40^o^C | | | | | |
| q_m_, mg/g  K_L_, L/mg  q_mI_/q_mn_  K_LI_/K_Ln_  R^2^  ARE  SSE  λ^2^  RMSE  HYBRID  SAE | 55.31^e^  0.024^e^  -  -  0.963^e^; 0.895*^e^  6.760  25.098  1.086  1.771  20.423  12.085 | 76.34  0.013  1,38  0.54  0.969  8.186  91.903  2.306  3.389  42.719  20.353 | 55.56  0.023  1  0.96  0.963  6.836  27.199  1.090  1.844  19.089  13.327 | 57.74  0.021  1.04  0.88  0.776  6.427  27.618  1.038  1.858  18.079  12.780 | 65.93  0.017  1.19  0.71  0.776  6.430  47.634  1.338  2.440  24.197  14.592 |
| F-300 pH6 T=30^o^C | | | | | |
| q_m_, mg/g  K_L_, L/mg  q_mI_/q_mn_  K_LI_/K_Ln_  R^2^  ARE  SSE  λ^2^  RMSE  HYBRID  SAE | 62.91^e^  0.047^e^  -  -  0.982^e^; 0.908*^e^  4.417  19.681  0.597  1.568  10.212  11.088 | 68.97  0.037  1.10  0.79  0.993  3.894  36.003  0.774  2.121  13.315  12.752 | 61.73  0.048  0.98  1.02  0.993  4.547  20.742  0.647  1.610  10.975  11.263 | 65.24  0.042  1.04  0.89  0,939  4.005  22.837  0.574  1.689  9.513  11.608 | 67.05  0.039  1.07  0.83  0.939  3.933  27.912  0.644  1.868  10.814  12.191 |
| F-300 pH6 T=40^o^C | | | | | |
| q_m_, mg/g  K_L_, L/mg  q_mI_/q_mn_  K_LI_/K_Ln_  R^2^  ARE  SSE  λ^2^  RMSE  HYBRID  SAE | 59.16^e^  0.045^e^  -  -  0.980^e^; 0.900*^e^  4.438  18.818  0.586  1.534  10.065  10.757 | 65.79  0.035  1.11  0.78  0.992  4.027  39.762  0.865  2.229  15.175  12.878 | 57.47  0.048  0.97  1.07  0.992  4.785  20.700  0.681  1.608  11.632  11.343 | 61.46  0.040  1.04  0.89  0.935  4.192  22.386  0.574  1.673  9.614  11.619 | 63.31  0.038  1.07  0.84  0.935  4.103  28.003  0.656  1.871  11.187  12.155 |
| * Determination coefficient R^2^ for the linear version of the Freundlich isotherm  ^e^ Data provided in the article by Lach 2024 | | | | | |
